# Supplementary material for: Addressing Microaggressions in Academic Health: A Workshop for Inclusive Excellence
Source: MedEdPORTAL. 2021 Feb 11;17:11103. doi: 10.15766/mep_2374-8265.11103 (PMC7880252; doi:10.15766/mep_2374-8265.11103)
Supplement: Supplementary file 1 — Cases & Facilitator Guides.docxPowerPoint.pptxTimetable for Learning Activities.docxHandouts for Learners.docxCore Definitions.docxPre- & Posttest.docx [file mep_2374-8265.11103-s001.zip › A. Cases & Facilitator Guides.docx]

**APPENDIX A**: Facilitator Pre-Work and Cases and Accompanying Facilitator Guides

***Pre-work for Facilitators***

The Microaggressions Triangle Model was developed by Ackerman-Barger and Jacobs as a framework for understanding microaggressions from a humanistic standpoint. This article would be an excellent resource for facilitators to read prior to holding their first discussion session. The scenarios and discussions in this toolkit were designed to get the learners to put themselves in the shoes of each person in the microaggression triangle [See Figure 1 Microaggressions Triangle Model]. A key concept that learners should take away from discussions about the cases is that there are multiple ways that any given situation can be experienced. There is no algorithm for how to respond a microaggression, but the Microaggressions Triangle Model provides a framework for making decisions about how to handle microaggression when the goal is to uphold principles of inclusion. The discussions about the cases are not about taking sides or deciding who is right or wrong, but to consider how an interaction could be moved forward to re-establish and restore relationships. The model focuses on three primary roles in microaggressions, the recipient, the source and the bystander.

**The Recipient** is the individual(s) who is on the receiving end of a microaggression. Recipients often feel conflicted about whether and how to respond to microaggressions. They are in a nearly impossible situation risking passively allowing misconceptions about their identity to remain or risking a confrontation that could easily backfire. Recipients have to make a decision to respond or not respond at precisely a time when the activity of their brain has been directed to the amygdala (the emotions-based area of the brain) versus the pre-frontal cortex (the area of the brain which organizes higher thinking and decision making). From the literature we know that receiving microaggressions can result in anxiety and depression, decreased self-esteem, and withdrawing for self-protection.^13^ Of the individuals involved in the microaggression, the recipient likely has the highest cognitive burden and should not be expected to have to be the one to confront the source.

**The Source:** For the purposes of this toolkit the source refers to the individual or individuals who are the source(s) of the microaggression. When a source realizes that they were responsible for a microaggression they may feel shame and humiliation which leads to a strong desire to defend themselves by justifying what they said or done. Justification is commonly hidden behind phrases such as “they are being too sensitive” or “I would never do that, because I’m not racist.” Yet microaggressions are not about the intent of the source, but about the interpretation of the receiver. Perceptions about the meaning of reality is socially constructed and varied according to temporal and cultural locations.^14^ In other words, how one interprets an encounter will be based on past experience and teachings- so no two interpretations will be the same. This signifies the importance of examining all roles in a microaggression encounter if the intention is to acknowledge the situation and repair relationships.

Microaggressions affect not only the recipient and the source, but bystanders and the overall campus climate**. The Bystander(s)** is the individual or are individuals who, though, not directly involved in the interaction, were present and aware of the interaction. The role of the bystander is difficult in that they may worry about becoming involved, becoming a target themselves, or making the situation worse. However, if you are a bystander, you are already involved. According to Coloroso^15^, doing nothing is not neutral, silence has meaning. “Active” bystanders are individuals who intervene in the situation. Many bystanders can relate to what it feels like receive a microaggression or to be the source of a microaggression and this perspective can be valuable in intervening in a way the upholds principles of inclusion. The role of bystanders is crucial to setting the tone of what is acceptable and unacceptable to the community.

The Microaggressions Triangle model provides a framework with acronyms to help individuals respond to microaggressions whether they are the recipient, the source or they bystander. Review PowerPoint slides prior to facilitation. Slides 10-15 in the PowerPoint presentation contain more in-depth explanations for how to implement the ACTION, ASSIST, and ARISE responses [see below and the PowerPoint in Appendix B].

**Microaggressions Triangle Model**

Recipient

ACTION

ACTION

Bystander

ARISE

ARISE

Source

ASSIST

ASSIST

| **Recipient-ACTION Approach^1^** | **Source-ASSIST Approach** | **Bystander-ARISE Approach** |
| --- | --- | --- |
| **A**sk a clarifying question | **A**cknowledge your bias | **A**wareness of microaggression |
| **C**ome from curiosity | **S**eek feedback | **R**espond with empathy) avoid judgment) |
| **T**ell what you observed | **S**ay you are sorry | **I**nquiry of facts |
| **I**mpact Exploration | **I**mpact, not Intent | **S**tatements that start with ‘I” |
| **O**wn thoughts and feelings | **S**ay **T**hank You | **E**ducate and **E**ngage |
| **N**ext steps |  |  |

Seven scenarios based upon student accounts of their experiences as well as scenarios crafted by the authors allowed learners to apply their knowledge [See Appendix A Cases & Facilitator Guides]. Each scenario was reviewed by multiple health professions audiences including nursing, medicine and physician assistant who were faculty, medical residents or graduate students. These individuals represented a wide range of racial/ethnic backgrounds, ages, genders and included members of the LGBT community. These reviews added depth to the discussion guide and helped the authors see past our own individual lenses.

| **Case 1: They Haven’t Kicked You Boys Out Yet?**  Consider the story of an incident as retold by two different students at two different interviews, unbeknownst to the other student. Rashid and Daevion are two first year medical students. They are the only two Black men in their cohort. They each described an interaction with a White male professor.  “So, me and a fellow classmate, who is also Black, were coming in early to get some last minute studying in before finals. And we ran into a professor that we had in Block 1. And I held the door for him, said, ‘Hi, how’s it going’ and then he’s like, ‘Oh, hi, how are you boys doing? They haven’t kicked you out yet?’ I didn’t know… was it unintentional or did it have deeper meaning? And me and my friend looked at each other and were in shock a little bit and didn’t know how to address it.  But we just went on and studied. But it was interesting because microaggressions don’t usually get to me but like I think with this one I was like, ‘Man!’, like, for 10 minutes instead of studying I was like, Ugh! Did he really just say that?” *Daevion*  “There was a time when I saw a previous professor who I consider a friend and I think he considers me a friend as well. And me and a classmate, he’s also African-American, were walking in the building. And he was like, ‘Oh, they haven’t kicked you all out yet?’ We’re friends, like, sarcasm whatever… But to somebody who isn’t like me, they could have easily been hurt by that, struck by that, triggered by that. And a bit of me was too. I was kinda like, ‘what?’”  *Rashid* |
| --- |

**Discussion Guide**

**Case 1: They Haven’t Kicked You Boys Out Yet?**

1. Who was the recipient of the microaggression? What aspect of the recipient’s identity was the target of the microaggression?

*Daevion and Rashid were the recipients of the microaggression. Their identity as Black Men in medical school was targeted.*

1. Who was the source of the microaggression? What is the direction of the hierarchy in the relationship between the recipients and the source? For example, was the source in a position of authority, or was their position lateral or subordinate to the recipient?

*The source was a professor who is in a position of authority to students.*

1. What was the nature of the microaggression? How it could have hurtful impact on the recipient?

*This interaction falls into the category of a microaggression because it was subtle and left the recipients wondering what to think about the interaction. It could be argued that the professor was simply joking with and trying to connect with the two students. It could be that the professor understood he was referencing race and history, but thought his relationship previous to the incident gave him social “permission” to “joke” or say what he said. Remember, as much as folks like to talk about* ***Intent,*** *the source has to take responsibility for and learn from the* ***Impact*** *(as experienced and expressed by the recipient, not the source) as well, intended or not. However, this joke, with these two students has historical, structural and cultural context that the professor either was oblivious to or did understand the relevance of.*

1. What is the historical, structural, cultural context of the microaggression?

There are several layers of microaggression to bring forward if learners do not identify them during the discussion.

*Key Points:*

- 1. *The use of the term “boys” in reference to men is diminishing and dehumanizing. The term boy was an expected part of the social order of the Jim Crow south. Whites did not need to call Blacks with a prefix (Mr., Miss), they did not have to recognize the last names, if the Black folks had one during slavery (see graves with only first names. Slavery was justified by scientific racism that held that Blacks were always childlike, and this slavery was an optimal human condition for Blacks because they needed [sic] that level of supervision from Whites. The use of the term “boy” in reference to grown men who were slaves in the U.S. gives the term a racialized context.*
  2. *Structural inequities have disproportionately excluded people of color in higher education. There are both overt and tacit assumptions that underrepresented people of color are under-prepared, under-motivated and under-qualified for medical school. Further, in the not so distant past there were efforts to scientifically prove that Blacks are intellectually inferior to whites [See Carl Brigham 1922. Brigham developed the Scholastic Aptitude Test in 1926 from which most standardized exams are modeled]^1^ So, when Daevion and Rashid question whether he has really been expecting them to be kicked out or to fail they are not being oversensitive.*
  3. *Although the professor may have been unaware of how this comment would be received by Rashid and Daevion, this is a problematic because many White people have not valued the importance of Black History, whereas Black people are very versed in White History and cultural norms.*

1. How might the recipient and source be viewing the situation differently? [This section is critically important in the triangle model. Do not try to establish who is right or wrong, or what should have been said or done. Instead, focus on each person involved and, given that people are complex and not all good or all bad, try to understand what their world view may be. This helps you make an informed decision about how to proceed].

*One of the confusing aspects of microaggressions is that different people in the same interaction might be having very different experiences. It is likely that while Rashid and Daevion feel attacked, the professor thinks that they were engaged in friendly banter.*

| **Recipient-ACTION Approach^2^** | **Source-ASSIST Approach** | **Bystander-ARISE Approach** |
| --- | --- | --- |
| **A**sk a clarifying question | **A**cknowledge your bias | **A**wareness of microaggression |
| **C**ome from curiosity | **S**eek feedback | **R**espond with empathy) avoid judgment) |
| **T**ell what you observed | **S**ay you are sorry | **I**nquiry of facts |
| **I**mpact Exploration | **I**mpact, not Intent | **S**tatements that start with ‘I” |
| **O**wn thoughts and feelings | **S**ay **T**hank You | **E**ducate and **E**ngage |
| **N**ext steps |  |  |

1. Discuss responses from each member involved in the interaction that could repair and re-establish relationships and restore reputations
   1. **Recipient (ACTION Approach):** Given the circumstance what are ways the recipient could address this (including not addressing it in the moment)? What can the recipient do to maintain and restore their well-being after this microaggression?

***In the moment:***

“Good morning Dr. XYZ, that sounded like you are surprised we are still here? Is that true?”

***Addressing it later:***

Hey, Dr. XYZ, remember when we ran into each other yesterday. I have been thinking about that. When you said, ‘they haven’t kicked you boys out yet’ I felt like you have been expecting us to drop out of school. I wanted to clarify that interaction with you.”

***If he is open to dialogue you could follow up with:***

“I am not sure if you know this, but given the history of the ‘boy’ in reference to African-American men- that word kinda falls on us wrong.”

- 1. **Source (ASSIST Approach):** Put yourself in the role of the source. Imagine that you become aware of your microaggression. What could you say or do to repair and re-establish relationship your relationship with the recipient (s)?

***Example response:***

“Daevion, Rashid, when I made that joke, I thought I was being friendly and funny- but now that I reflect on it I see that it was not funny and I am really sorry. Will you give me a chance to do better in the future?”

- 1. **Bystander (ARISE Approach):** Whether there were bystanders or not in this scenario consider the role of a bystander or bystanders. How could they address this situation? Should it be done now or later?

What could be said or done to support or protect the recipient?

***Peer (of students) bystander***

“Hey, Rashid, Daevion, I saw that interaction that happened with you and Dr. XYZ in the doorway yesterday. I left feeling uneasy. How are you two doing?”

What could be said to the source that could help them become aware of the microaggression while keeping them engaged in the interaction? What are ways to help the source re-establish their relationship with the recipient (s) and restore or protect their reputation?

***Colleague of Dr. XYZ bystander***

“I wanted to follow up with you about an interaction I saw yesterday. When Rashid and Daevion were holding the door open for you, you expressed surprise that they hadn’t been kicked out yet and you called them boys. I noticed their faces drop. I am not sure if you were joking, but if you were, I think it may not have come out like you thought. I wanted to let you know because we have worked together for a while and that statement does not align with who I know you to be.”

1. Lemann N. *The Big Test: The Secret History of American Meritocracy.* 1999.

2. Cheung F, Ganote C, Souza T. *Microaggressions and microresistance: Supporting and empowering students* 2016.

**Case 2: Taboo to Touch**

**From a Student Interview:**

There was a microaggression that almost made me fail out of school. I was doing my pediatric rotation. I had a little baby, a newborn, who was not doing well from a Hispanic or Latino family. We have this thing called “Mollera”, the front fontanelle, the soft part at the front of the head. We believe if you press on it or if you shake the baby too much, well, it’s super taboo for us to touch it. It’s part of the nursing physical assessment to palpate fontanelle. I went in with my preceptor and she did it. I was paying attention to the family because I knew what that meant for us. Since the baby was already not in the best health, their facial expression was one of almost terror- like, “Why are you touching my baby there?” But they didn’t say anything. So, when my preceptor said, “Now you do it.” I said, “you know actually … I think that’s okay. I’ll do the other things. I can explain to you about it after”. I did the rest of the assessment and when we walked out, I tried to explain to her how it was taboo and that she had already done it. I was trying to have a patient centered approach to my care. I got slammed for it. She was actually very upset with me that I had disobeyed her in front of the patient’s family. From that point on, it was just horrendous my experience with her. I almost failed that rotation.

I ended up talking to my department head. However, when I went to talk to them, they were already aware because the preceptor had called and said I wasn’t doing as I was told. I was told to suck it up. At our School of Nursing, if you fail the class, you fail the year. You repeat. There is no remediation for any clinical or any class that we do. So, the pressure is immense to pass. What ends up happening is that we get these preceptors who are very insensitive to culture and diversity. When we speak out, (I’m not the only student who has experienced this) we tell each other. The students of color at the School of Nursing warn each other about certain preceptors, about saying certain things. So, we have it as culture within our school that we warn each other because every year one minority student of the entering class fails out. It’s usually because of a preceptor and it usually because of the same preceptor all the time.

**Discussion Guide**

**Case 2: Taboo to Touch**

1. Who was/were the recipient(s) of the microaggression? What aspect of the recipients’ identity was the target of the microaggression?

*The student was the recipient of a microaggression, and it could be argued that the family was too.*

- *The Latino/Hispanic identity was a target.*
- *The student role may have been a target*
- *The patient role may have been a target*

1. Who was the source of the microaggression? What is the direction of the hierarchy in the relationship between the recipients and the source? For example, was the source in a position of authority, or was their position lateral or subordinate to the recipient?

*The source was the preceptor/nurse who was in a significant power position, such that she could influence a grade of failure and in fact, according to the student account, put the student in some academic jeopardy. She also possessed authority as the healthcare provider to the infant and family.*

| *Preceptor*  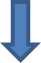  *Nursing Student* | *Nurse*  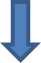  *Family/Patient* |
| --- | --- |

1. What was the nature of the microaggression? How it could have hurtful impact on the recipient?

*The preceptor violated a cultural custom (likely unknowingly) then diminished and rejected the students experience of and expertise in this matter. This may fall into the category of a micro-invalidation where the experiences of groups of people are undervalued, dismissed and ignored.*

1. What is the historical, structural, and/or cultural context of the microaggression?

*Key Points*

- 1. *Historically people of Latinx or Hispanic ancestry have been marginalized, underserved and “otherized” in the U.S. Many of the lands in the U.S. belonged to Mexico and were violently appropriated by the U.S. This dynamic continues in our politics as undocumented individuals are often fearful of deportation and separation from their families.*
  2. *The structure and culture of healthcare in the U.S. is a Western approach to medicine which focuses on physiology, pharmacology, and invasive intervention; often dismissing spiritual and cultural practices. We also approach healthcare in a paternalistic way meaning that patients and families are treated as recipients of care rather than partners in care.*

1. How might the recipient and source be viewing the situation differently? [This section is critically important in the triangle model. Do not try to establish who is right or wrong, or what should have been said or done. Instead, focus on each person involved and, given that people are complex and not all good or all bad, try to understand what their world view may be. This helps you make an informed decision about how to proceed].

***The student*** *seems to have viewed her relationship with the preceptor (perhaps erroneously) as a partnership with an egalitarian component. Patient-centered care is a central theme in many nursing schools where students are encouraged to advocate for their patients. This is the approach that the student seemed to have intended. The student, having knowledge and potentially in-group status with the family may have felt obligated to serve as an ambassador- torn between her role as a student and her membership in this group.*

***The family*** *was likely focused on and concerned for the well-being of their newborn baby. The family may not have known the roles or relationships of the nurse and nursing student. If they recognized that the student was of a similar background, there may have been a hope that she would intervene.*

***The preceptor*** *is both a woman and a nurse which may provide some insight into how she is experiencing the situation. She is likely feeling contradicted by the student. Historically the knowledge of both women and nurses is often questioned and discredited. Whether this is occurring or not during the moment, it is likely she has experienced students or others interjecting or undermining the care she has provided, and she may be very well attuned to these types of situations and reacting accordingly in this moment.*

| **Recipient-ACTION Approach^1^** | **Source-ASSIST Approach** | **Bystander-ARISE Approach** |
| --- | --- | --- |
| **A**sk a clarifying question | **A**cknowledge your bias | **A**wareness of microaggression |
| **C**ome from curiosity | **S**eek feedback | **R**espond with empathy) avoid judgment) |
| **T**ell what you observed | **S**ay you are sorry | **I**nquiry of facts |
| **I**mpact Exploration | **I**mpact, not Intent | **S**tatements that start with ‘I” |
| **O**wn thoughts and feelings | **S**ay **T**hank You | **E**ducate and **E**ngage |
| **N**ext steps |  |  |

1. Discuss responses from each member involved in the interaction that could repair and re-establish relationships and restore reputations
   1. **Recipient (ACTION Approach):** Given the circumstance what are ways the recipient could address this (including not addressing it in the moment)? What can the recipient do to maintain and restore their well-being after this microaggression?

*In this case the student has tried several mechanisms to address this situation and really needs a support person. It may not be safe for her to address this with the preceptor. She believes that her status as a student is in jeopardy. Not only could this derail her career and negate the years she has invested thus far, but most students are accruing student loan debt which could be catastrophic for a student financially if they are unable to secure a position as a healthcare provider.*

*From this scenario, it is unclear how the student phrased the feedback to the preceptor.*

***Ideas for discussing with the preceptor***

*I wanted to let you know that I really appreciate the time and effort you invested in me and I didn’t mean to seem unwilling to help or ungrateful [this acknowledges how the source may have interpreted the situation], I just wanted to let you know about a cultural taboo within my community that not everyone knows about. Would it be okay if I share it with you?*

***Ideas for discussion with the school***

*From the perspective of the school, nursing leaders tend to worry about professionalism and because clinical placements are scarce, they may be eager to maintain all clinical placements and preceptors.*

*“I wanted to share an experience I had today in clinical which don’t align with our mission statement about diversity and inclusion and does not align with what we have been taught about patient-centered care. I know there is a lot at stake here, but maybe you could hear me out and we could strategize next steps that could help me be successful in this rotation and make sure the school maintains its relationship with the hospital and the community.”*

- 1. **Source (ASSIST Approach):** Put yourself in the role of the source. Imagine that you become aware of your microaggression. What could you say or do to repair and re-establish relationship your relationship with the recipient (s)?

***Preceptor to student***

*“I am sorry, I am reacting badly to your feedback. I felt like you were questioning my care and skills. It seems like this has been happening to me a lot lately. I know you were trying to tell me something about the family. I am sorry I behaved that way. What were you trying to say?*”

- 1. **Bystander (ARISE Approach):** Whether there were bystanders or not in this scenario consider the role of a bystander or bystanders. How could they address this situation? Should it be done now or later?

*Because all of the individuals in the room were involved in the interaction it can be argued that there were no bystanders in this situation. It could be argued that the student witnessed a microaggression or insensitivity pointed at the family and therefore was a bystander to that interaction.*

*In this case consider the school as the bystander. What could the school do to support or protect the student?*

***First, validate the student’s feelings****. “Wow, that sounds like a really uncomfortable situation. I am sorry you had to go through that.”*

***State the facts,*** *“This is a tough situation, our clinical relationships are critical to our ability to get students hands on experience but supporting students and providing patient-centered care is a core value for us.”*

***State what the school can do,*** *“We need to do some investigating about this issue. It may take a little bit of time, but we will reach out to you in the next week or so to see how you are doing and to follow-up on this case. Meantime, we have multiple campus services which may be helpful to you as you process what happened [provide resources].*

- *What could be said to the source that could help them become aware of the microaggression while keeping them engaged in the interaction? What are ways to help the source re-establish their relationship with the recipient (s) and restore or protect their reputation?*

*It would make sense for the school to reach out to the manager of the hospital unit, who would, then, be the ideal person to speak with the source.*

*Ideas for how to frame that conversation.*

*“Hi, XYZ, thank you for meeting with me today. I wanted to check in with you about a phone call that I had from ABC school. We have had a long time relationship with the school and this community connection is important to us, as is the well-being of both the students that rotate through here and staff such as yourself who care for our patients. I understand that you worked with a student last week who did not palpate fontanels during as assessment. Her perception was that you were upset with her about it. I hoped to hear your side and share with you the perspective of the student.” [Listen to XYZ- this is a crucial time to understand her perspective and frame how you can both support her and help her learn and grow as an individual who cares for Latinx patients and who works with students]*

1. Cheung F, Ganote C, Souza T. *Microaggressions and microresistance: Supporting and empowering students* 2016.

**Case 3:  Invite Your Husband**

Dr. Sheila Williams has just been hired as a new assistant professor and is meeting the other faculty at a departmental faculty meeting on her first day. Dr. Williams has just moved to town with her family and is asking the other faculty members about what to do in town with young kids. Dr. Arroyo suggests the children’s museum in town. Dr. Jensen, wishing to make her new colleague feel welcomed and included, asks Dr. Williams, “I’d like you and your husband to come over for dinner next weekend. You can bring your kids and your family can meet my family. I’m sure our husbands will get along very well.” Dr. Williams is not sure what to say because she has a wife, not a husband. She decides to take a chance and let Dr. Jensen know that she is married to a woman. Dr. Jensen responds to this by saying replies, “No need to be defensive. I’m not homophobic. I have a lot of friends who choose that lifestyle.”

**Discussion Guide**

**Case 3- Invite Your Husband**

1. Who was the recipient of the microaggression? What aspect of the recipient’s identity was the target of the microaggression?

*The recipient of the microaggression was Dr. Williams, based on her identity as a lesbian.*

1. Who was the source? What is the direction of the hierarchy in the relationship between the recipients and the source? For example, was the source in a position of authority, or was their position lateral or subordinate to the recipient?

*The source of the microaggression was Dr. Jensen. Her role is not clear, but we can assume that she is an established faculty member while Dr. Williams is new.*

1. What was the nature of the microaggression? How it could have hurtful impact on the recipient?

*The first microaggression is the heteronormative assumption that Dr. Williams would have a husband. Dr. Williams could be single, or she could have a wife, as is the case in this scenario. This comment is hurtful to Dr. Williams because it gives the message that a woman should be married to a man, and that anything outside of this is abnormal and maybe even wrong. It also gives the implicit message that since she has kids, she should be married. Given that Dr. Williams is new, this type of comment could be particularly hurtful because she does not yet know if the culture of this department is inclusive of the LGBTQ community and whether it would be safe for her to come out. She also does not know anything about Dr. Jensen’s cultural or religious beliefs, which could inform her values about sexuality. Explicit and implicit anti-gay bias is prevalent. The microaggressions escalated when Dr. Jensen suggested that Dr. Williams was being “defensive” when she was simply letting her know that she had a wife instead of a husband, and when Dr. Jensen talked about Dr. Williams’ “lifestyle” being a “choice.”*

1. What is the historical, structural, cultural context of the microaggression?

*Key Points*

- 1. *There are approximately 9 million people in the United States who identify as LGBT. Although acceptance of LGBT people is improving in the United States, explicit and implicit anti-gay bias remains prevalent.*
  2. *In one study of first year medical student across the county almost half (45.8%) were found to have explicit anti-gay bias and most (81.5%) had at least some implicit anti-gay bias. ^1^*
  3. *In 2011, The Institute of Medicine released a report on the depth and breadth of unique health disparities in the LGBT community. These include both physical and mental health outcomes, experiences of violence, as well as an exploration of barriers to healthcare.^2^*
  4. *In the not so distant past homosexuality was not only stigmatized but was categorized as a mental illness in the Diagnostic and Statistical Manual of Mental Disorders up until 1973.  Transgender status was considered a “gender identity disorder” in the DSM until 2013, when it was changed to “gender dysphoria”. ^3^*
  5. *Ilan Meyer, in discussing the experiences of the LGBT community, coined the term ‘minority stress’ which explains how stigma, prejudice, and discrimination create a hostile and stressful social environment that causes mental health problems. The model describes stress processes, including the experience of prejudice events, expectations of rejection, hiding and concealing, internalized homophobia, and ameliorative coping processes.^1^*

1. How might the recipient and source be viewing the situation differently?  [This section is critically important in the triangle model. Do not try to establish who is right or wrong, or what should have been said or done. Instead, focus on each person involved and, given that people are complex and not all good or all bad, try to understand what their world view may be. This helps you make an informed decision about how to proceed]

| **Recipient-ACTION Approach^4^** | **Source-ASSIST Approach** | **Bystander-ARISE Approach** |
| --- | --- | --- |
| **A**sk a clarifying question | **A**cknowledge your bias | **A**wareness of microaggression |
| **C**ome from curiosity | **S**eek feedback | **R**espond with empathy (avoid judgment) |
| **T**ell what you observed | **S**ay you are sorry | **I**nquiry of facts |
| **I**mpact Exploration | **I**mpact, not Intent | **S**tatements that start with ‘I” |
| **O**wn thoughts and feelings | **S**ay **T**hank You | **E**ducate and **E**ngage |
| **N**ext steps |  |  |

*Dr. Jensen was likely trying to be hospitable to the new faculty member by inviting their families to spend time together, and she was likely trying to make Dr. Williams feel welcome in the department. Her second comment about “having a lot of friends who choose that lifestyle” was likely meant to demonstrate that she is not biased or homophobic. However, Dr. Williams is very offended by these comments, which show an underlying assumption of heteronormativity and a belief that homosexuality is a choice and simply a lifestyle. Dr. Jensen’s comments also assume that if you have friends in a particular group, this exempts you from having bias toward that group, which is not true.*

1. Discuss responses from each member involved in the interaction that could repair and re-establish relationships and restore reputations

1. **Recipient (ACTION Approach):** Given the circumstance what are ways the recipient could address this (including not addressing it in the moment)? What can the recipient do to maintain and restore their well-being after this microaggression?

*In this public situation, and since Dr. Williams is new, it would be better for her to address the microaggressions after the faculty meeting and in private with Dr. Jensen. At this time, Dr. Williams could use ask Dr. Jensen what she means by her comments, such as “What do you mean when you say you have friends who choose that lifestyle?” She can also come from curiosity and not judgement, as in not assuming that Dr. Jensen meant to be offensive with her comments. She could tell Dr. Jensen how she felt about her comments, as in “When you invited me and my family to your home I felt very welcomed but your assumption that I had a husband made me feel uncomfortable.” She could share the impact of Dr. Jensen’s statements and how she feels by saying, “You seem like a very thoughtful and welcoming person but the assumption that I had a husband made me wonder if it is safe for me to be out in this culture.” Depending on how this conversation goes, next steps could include Dr. Williams taking about bringing her wife to the dinner and how Dr. Jensen and her family would feel about that.* *However, if Dr. Jensen continued to be as defensive and hurtful as she was during the original interaction, Dr. Williams could consider talking with the department Chair about the incident and/or reporting Dr. Jensen.*

1. **Source (ASSIST Approach):** Put yourself in the role of the source. Imagine that you become aware of your microaggression. What could you say or do to repair and re-establish relationship your relationship with the recipient (s)?

*Dr. Jensen could acknowledge her bias by saying, “You’re right, I assumed that you would have a husband.” She could also acknowledge her bias around her statement that being gay is a “choice” and a “lifestyle.” She could then ask Dr. Williams for feedback on how and why she experienced her statements as offensive, and apologize to her once she understood that the impact of her comments did not match her intent of being welcoming. Thanking Dr. Williams for sharing her reactions would demonstrate Dr. Jensen’s emotional intelligence and humility, and it committing to work on her bias would be a great way to rebuild their relationship.*

1. **Bystander (ARISE Approach):** Whether there were bystanders or not in this scenario consider the role of a bystander or bystanders. How could they address this situation? Should it be done now or later?

- What could be done to support or protect the recipient?
- What could be said to the source that could help them become aware of the microaggression while keeping them engaged in the interaction? What are ways to help the source re-establish their relationship with the recipient (s) and restore or protect their reputation?

*Dr. Arroyo, another faculty member with unknown status in the hierarchy, is one bystander in this case. Given the sensitivity of the situation, it would be best for him to address it later with Dr. Jensen and also with Dr. Williams. Dr. Arroyo could say to Dr. Jensen, “Earlier today in the department meeting, I noticed that you were kind enough to invite our new faculty member to meet your family, but the look on her face when you mentioned how your husbands would get along very well made me worry. I know you to be a thoughtful and welcoming person but your assumption that she would have a husband could come off as heteronormativity or even anti-gay bias, and I assume that was not your intent. I’m sharing this with you because I know you to be a person who values equity and inclusion, and I would hate for you to inadvertently make anyone feel unwelcomed or uncomfortable. I’m wondering how you could engage with Dr. Williams in a way that is consistent with your values?”*

*Dr. Arroyo could also debrief the situation later with Dr. Williams, to talk with her about her responses and to validate her feelings, to ensure that she feels understood and supported, and to assure her that she is welcome and safe in the department.*

1. Meyer I. Prejudice, social stress, and mental health in lesbian, gay and bisexual populations: Conceptual issues and research evidence *Psychology Bulletin* 2003;129(5):674-697.

2. *The Health of Lesbian, Gay, Bisexual and Transgender People: Building a Foundation for Better Understanding* Washington DC: Institute of Medicine (US). Committee on Lesbian, Gay, Bisexual and Transgender Health Issues and Research Gaps and Opportunities 2011.

3. Understanding the health needs of LGBT people. National LGBT Health Education Center Published 2016. Accessed.

4. Cheung F, Ganote C, Souza T. *Microaggressions and microresistance: Supporting and empowering students* 2016.

**Case 4: Don’t Worry, You’ll Get In**

Dr. Wallace is leading a small group advising session for medical students who are applying for residency programs in OB/GYN. As this specialty is getting more competitive, the medical students in the group are quite anxious and are discussing their worries about whether they will be matched up with a desired program. Maria, a Latina medical student who is a first-generation college graduate, is telling the group how she applied to more residency programs than the national average because she is especially worried about not getting into this competitive specialty. Caleb, a fellow medical student who is a White male, assured Maria that she didn’t have to worry about getting into an OB/GYN residency program because she is Latina and there is a shortage of Latinxs, so she would be sure to get in as a diversity hire. Dr. Wallace noticed that Maria and several other students in the group were looking frustrated and that the tension in the room was high. Dr. Wallace didn’t say anything because she was not sure how to respond, instead she changed the topic and moved along to interviewing techniques.

**Discussion Guide**

**Case 4: Don’t Worry, You’ll Get In**

1. Who was the recipient of the microaggression? What aspect of the recipient’s identity was the target of the microaggression?

*Maria was the recipient of Caleb’s microaggression, based on her identity as a Latina. Furthermore, Dr. Wallace’s failure to address Caleb’s offensive comment was also a microaggression.*

1. Who was the source of the microaggression? What is the direction of the hierarchy in the relationship between the recipients and the source? For example, was the source in a position of authority, or was their position lateral or subordinate to the recipient?

*Caleb was the source of the microaggression. As a fellow student, Caleb’s position was lateral to that of the recipient.*

1. What was the nature of the microaggression? How it could have hurtful impact on the recipient?

*Caleb’s comment that Maria would get into an OB/GYN residency because of her race was meant to reassure her not to worry about how competitive the specialty is, but it was a microinvalidation because it gave the message that she would get into the residency not because of her merit but because of her race. This is hurtful to Maria, who has worked as hard as anyone else go get through medical school and be competitive for this specialty because of her high grades and Step scores. It is likely that Maria has heard this type of comment before, with the assumption that she got into college and then medical school because of her race and policies like affirmative action. Furthermore, this type of situation could reinforce Maria’s stereotype threat. If Maria believes that her behaviors are being seen through the lens of racial stereotypes by residency interviewers, in the same way that she perceives Caleb viewing her, the quality of her interviews could be threatened.*

1. What is the historical, structural, cultural context of the microaggression?

*Key Points*

- 1. *The influence of eugenics in science and education in the early to mid- twentieth century continues to influence commonly held notions about intellectual capability today. To understand the heritage of the United States in this regard refer to a book titled, The Ascription of Intelligence written by Carl Brigham in 1923 who later created the scholastic aptitude test (SAT).^1,2^ Brigham attempted to stratify intellect based on race with Northern Europeans being intellectual elites, then intellect decreasing as the geography moved south through Italy and the Mediterranean, to Africa.*
  2. *Research shows that students who are African American/Black, Latinx and Native American are aware of negative stereotypes assumption about their groups not being as intellectually capable, qualified, motivated or prepared for academia. Ironically, fear of confirming these stereotypes can lead to a set of psychological circumstances that impede short term memory and the ability to process information which lower academic performance. This a phenomenon known as stereotype threat.^3^*
  3. *In addition to stereotypes, are misguided notions about affirmative action and admissions. The term affirmative action is first seen in an executive order by John F. Kennedy in 1961 which stated, “"take affirmative action to ensure that applicants are employed and that employees are treated during employment without regard to their race, creed, color, or national origin."^4^ The term “affirmative” action was meant to underscore that we needed to change our hiring practices and that employers needed to actively avoid discriminatory practices because this would not happen passively or by doing things the way we have always done them. The response to this order included both mandatory and voluntary steps. Although some entities attempted to use quotas to fulfill executive orders related to affirmative action, this was not and is not legal in the United States. However, many people believe that quotas are the underlying principle of affirmative action and misconstrue this to mean that unqualified women or applicants of color are selected for hire or admission over otherwise qualified white men. This spreads into an additional misconception that anyone who is a woman or an individual of color must have been hired or admitted to fill a quota rather than because of their merit and qualifications.*
  4. *Resources for learning the origins and meaning of affirmative action.*

[*Executive Order 10925*](https://www.eeoc.gov/eeoc/history/35th/thelaw/eo-10925.html) *^4^*

[*Executive Order 11246*](https://www.archives.gov/federal-register/codification/executive-order/11246.html)*- Equal Employment Opportunity ^5^*

[*Civil Rights Act of 1964-National Archives*](https://www.archives.gov/education/lessons/civil-rights-act) *^6^*

1. How might the recipient and source be viewing the situation differently? [This section is critically important in the triangle model. Do not try to establish who is right or wrong, or what should have been said or done. Instead, focus on each person involved and, given that people are complex and not all good or all bad, try to understand what their world view may be. This helps you make an informed decision about how to proceed].

*Caleb may have viewed his comment as a compliment and an assurance to Maria that she doesn’t have to worry so much about getting into a residency. However, Maria could be hurt by this comment because it invalidates how hard she has worked and assumes that she will get in because of her race and not her merit.*

| **Recipient-ACTION Approach^7^** | **Source-ASSIST Approach** | **Bystander-ARISE Approach** |
| --- | --- | --- |
| **A**sk a clarifying question | **A**cknowledge your bias | **A**wareness of microaggression |
| **C**ome from curiosity | **S**eek feedback | **R**espond with empathy (avoid judgment) |
| **T**ell what you observed | **S**ay you are sorry | **I**nquiry of facts |
| **I**mpact Exploration | **I**mpact, not Intent | **S**tatements that start with ‘I” |
| **O**wn thoughts and feelings | **S**ay **T**hank You | **E**ducate and **E**ngage |
| **N**ext steps |  |  |

1. Discuss responses from each member involved in the interaction that could repair and re-establish relationships and restore reputations
   1. **Recipient (ACTION Approach):** Given the circumstance what are ways the recipient could address this (including not addressing it in the moment)? What can the recipient do to maintain and restore their well-being after this microaggression?

*Maria could ask Caleb what he meant by his comment that she would get in because she is Latina. She could let him know that the comment he meant as a reassurance came off as offensive to her by saying, “Ouch! I know you are trying to make me feel better but your comment assumes that I will get in because of my race and not because of my merit.” If she feels safe, she could express her feelings about the comment, as in saying “Many people assume that I’m here just because of affirmative action, and that is painful because I have worked just as hard as anyone else.” If Caleb responds well, Maria’s next steps could involve offering to talk more about it later, which could help repair their relationship.*

- 1. **Source (ASSIST Approach):** Put yourself in the role of the source. Imagine that you become aware of your microaggression. What could you say or do to repair and re-establish relationship your relationship with the recipient (s)?

*Caleb could notice the discomfort of Maria and other students in the room after making his comment, and acknowledge that his comment was offensive. He could seek feedback from the uncomfortable students about why his comment was so offensive. For example, he could say, “I meant that as a reassurance but I noticed that you seemed upset by my comment. Can you help me to understand your reaction better? Upon understanding why his comments were so offensive, Caleb should apologize for the impact of his statement, even though his intent was so different. He could say, “Although it was my intent to reassure you, I can see how the comment would could be hurtful, and I am very sorry for that.” A commitment to work on his bias would go a long way in repairing his relationship with Maria and the other students in the group.*

- 1. **Bystander (ARISE Approach):** Whether there were bystanders or not in this scenario consider the role of a bystander or bystanders. How could they address this situation? Should it be done now or later?
- What could be done to support or protect the recipient?
- What could be said to the source that could help them become aware of the microaggression while keeping them engaged in the interaction? What are ways to help the source re-establish their relationship with the recipient (s) and restore or protect their reputation?

*As the leader in the room, Dr. Wallace could address the microaggression by modeling for the students in the room how to be a good ally and uphold health care’s values of equity and inclusion.* *She could say, “I noticed that people seemed upset when Caleb made that comment. Knowing Caleb as a kind person, I can guess that he was trying to be reassuring to Maria but I worry that his comment may have been made on a biased assumption that she would get in because of her race and not because of her merit and hard work. Many people of color face assumptions like this, and it can be exhausting and very invalidating.” She could follow up with Caleb and debrief by saying, “I know it can feel bad to inadvertently offend someone. We’ve all been there. The important thing is to own it, apologize to the group, and learn all you can from discovering that you have a blind spot.”*

*Dr. Wallace could also debrief with Maria to validate her feelings and ensure that she feels heard and understood, as this is likely not the first time she has encountered the bias that her success is due to affirmative action rather than her merit. In addition, since other bystanders in the room were also offended by Caleb’s students, Dr. Wallace could also debrief with them.*

1. Lemann N. *The Big Test: The Secret History of American Meritocracy.* 1999.

2. Rosales J. The Racist Beginnings of Standardized Testing. *NEA Today Magazine.* 2018. <http://www.nea.org/home/73288.htm>.

3. Steele C, Aronson J. Stereotype threat and the intellectual test performance of African-Americans *Journal of Personality and Social Psychology.* 1995;69:797-811.

4. Kennedy JF. Executive Order 10925. In: Government US, ed1961.

5. Executive Order 11246- Equal employment opportunity In: Government US, ed1965.

6. The Civil Rights Act of 1964 and the Equal Employment Opportunity Commission In: Government US, ed1964.

7. Cheung F, Ganote C, Souza T. *Microaggressions and microresistance: Supporting and empowering students* 2016.

**Case 5: A Day in the Life of Female Surgeons**

Juana is a 25-year-old Latina who is on her Surgery clerkship in her third year of medical school. For the past two weeks, Juana has been rotating with Dr. Linda Watson, who has inspired her to specialize in surgery and go into academic medicine. Today, she is rotating with Dr. Joaquin Hernandez, her new surgery attending, and they are scrubbing in for a case along with Dr. Arash Hakim, a surgery resident. Dr. Hernandez asks Juana what specialty she wants to go into, and Juana eagerly replies that she plans to go into surgery. Dr. Hernandez responds, “It must be really hard to be a surgeon and a mom though.” Juana talks about what a great role model Dr. Watson has been, especially around balancing her work and family life. Dr. Hernandez shrugs and replies, “Oh, I’m not saying it can’t be done, I’m just saying that every time Dr. Watson is in the OR and her girls have a soccer game or recital, she has some really tough choices to make.” Juana looks uncomfortably at Dr. Hakim and wonders to herself whether Dr. Hernandez has had any similar conversations with this resident, since fathers would likely have similar difficult decisions. Before anyone can say anything, Dr. Hernandez shrugs again and changes the topic of the conversation.

**Case 5: A Day in the Life of Female Surgeons**

**Discussion Guide**

1. Who was the recipient of the microaggression? What aspect of the recipient’s identity was the target of the microaggression?

*Juana was the recipient of the microaggression, based on her gender and possibly also based on her ethnicity as a Latina, given stereotypes of Latinos having large families.*

2. Who was the source of the microaggression? What is the direction of the hierarchy in the relationship between the recipients and the source? For example, was the source in a position of authority, or was their position lateral or subordinate to the recipient?

*The source was Dr. Hernandez, the attending, who is in a position of power and authority over Juana. Dr. Hernandez evaluates all students, like Juana, and he may also have some power over her career, especially if she goes into Surgery.*

3. What was the nature of the microaggression? How it could have hurtful impact on the recipient?

*Dr. Hernandez is assuming that Juana will want a family and that she will experience problems balancing her work and family life as a surgeon. Based on her gender as a female, and possibly also based on her ethnicity as a Latina, he is trying to warn her that if she goes into surgery, she will have to make difficult decisions on a daily basis where she has to choose between her work and her children. It is unlikely that he has this type of conversation with male clerkship students who wish to pursue a career in surgery. His statements could have a hurtful impact on Juana by steering her away from a specialty about which she is excited and/or discouraging her from believing that she can have both a career and a family. The statements could give Juana the idea that surgery is not for females, especially those who want a family. Dr. Hernandez also assumes that all women want to have children, which is not always the case.*

4. What is the historical, structural, cultural context of the microaggression?

*Key Points*

- 1. *Although female medical students now outnumber males, sexual harassment and gender bias are prevalent in medicine. A recent report of the National Academies of Sciences Engineering and Medicine indicated that the prevalence of sexual harassment in academic medicine is almost twice that in other sciences or engineering, with 50% of female medical students experiencing sexual harassment from faculty or staff.^1^ As a result of sexual and gender harassment, victims often report higher levels of stress, burnout, dissatisfaction, and decrements in performance.*
  2. *Women in medicine also face significant gender bias. It is not uncommon for female doctors to be referred to as the nurse or medical assistant. Despite some recent studies showing that female physicians have better patient outcomes than their male counterparts^2^, doctors are less likely to refer patients to a female surgeon after a bad patient outcome whereas referrals to male physicians are unaffected by poor patient outcomes.^3^ Furthermore, after a bad experience referring to one female physician, doctors are less likely to refer to any female surgeons in the same specialty whereas this spillover effect does not occur with male physicians. And gender disparities exist in academic medicine as well, with female attendings receiving significantly lower clerkship teaching evaluations across four core clinical rotations than male counterparts.^4^ Female speakers at Grand Rounds are less likely to be introduced as “doctor” than are male speakers.^5^ Women in academic medicine are also not on parity with men at the levels of associate or full professor, and especially in leadership positions.^6^*
  3. *Even without the additional burden of sexual or gender harassment, female physicians face additional challenges compared with males. Unfortunately, Dr. Hernandez may be accurate in his statements to Juana. Women in medicine, even those who are in dual-physician households, carry a heavier burden of domestic duties, working on average nine more hours per week on domestic activities than male counterparts.^7^ Female physicians with children are also more likely to cut back professionally to manage domestic duties and to take time off when children are sick than male physicians. This work-family conflict, which is more significant for female doctors than males, is thought to account for the higher rates of depression in new doctors entering residency.^8^ A recent publication highlighted that almost 40% of female physicians cut back to part time or leave medicine altogether within 6 years of graduating from residency, in an effort to reduce work-family conflict.^9^ Many of these women were not simply choosing family over their careers, but felt they did not have a choice to balance both due to institutional barriers such as discrimination because of pregnancy or breastfeeding, leave policies, and challenges associated with reentry (i.e., needing a proctor or additional training after taking a few years off to care for a family member).*

5. How might the recipient and source be viewing the situation differently?

[This section is critically important in the triangle model. Do not try to establish who is right or wrong, or what should have been said or done. Instead, focus on each person involved and, given that people are complex and not all good or all bad, try to understand what their world view may be. This helps you make an informed decision about how to proceed].

*Dr. Hernandez may have thought he was be helpful to Juana, by sympathizing with how difficult it must be for women who want to go into surgery and possibly warning her away from this specialty. However, Juana likely sees this as a microaggression since Dr. Hernandez is likely having these conversations with female clerkship students only.*

*We also don’t know at this point whether Dr. Hernandez may have struggled with this as a father with small children at this may be his motivation.*

| **Recipient-ACTION Approach^10^** | **Source-ASSIST Approach** | **Bystander-ARISE Approach** |
| --- | --- | --- |
| **A**sk a clarifying question | **A**cknowledge your bias | **A**wareness of microaggression |
| **C**ome from curiosity | **S**eek feedback | **R**espond with empathy) avoid judgment) |
| **T**ell what you observed | **S**ay you are sorry | **I**nquiry of facts |
| **I**mpact Exploration | **I**mpact, not Intent | **S**tatements that start with ‘I” |
| **O**wn thoughts and feelings | **S**ay **T**hank You | **E**ducate and **E**ngage |
| **N**ext steps |  |  |

1. Discuss responses from each member involved in the interaction that could repair and re-establish relationships and restore reputations
   1. **Recipient (ACTION Approach):** Given the circumstance what are ways the recipient could address this (including not addressing it in the moment)? What can the recipient do to maintain and restore their well-being after this microaggression?

*Juana could choose not to address the attending’s comments in that moment, and she could talk with Dr. Hernandez later, in private. Alternatively, she could choose not to address the comments at all, as this is not her responsibility. Should she choose to address the attending’s comments, she could say something along the lines of, “I appreciate what you are saying but I’m not sure yet whether I want to have children. And, if I do, I’ll be sure to pick a partner that will participate equally in childcare duties. I want to be in a field that I am passionate about, and Surgery is where I want to be.”*

- 1. **Source (ASSIST Approach):** Put yourself in the role of the source. Imagine that you become aware of your microaggression. What could you say or do to repair and re-establish relationship your relationship with the recipient (s)?

*Dr. Hernandez could have used mindfulness, present-moment awareness, and perspective taking skills to notice the reaction on Juana’s face, and use values clarification to realize the discrepancy between his intention of helping Juana and the outcome of offending her. Also, he should have focused on the impact of the comment rather than the intention of helping Juana. A simple comment such as, “Oops!” could convey that he realized he made a mistake. Dr. Hernandez could have then taken responsibility for his assumptions, apologized for the comment, and expressed a commitment to change the behavior in the future. Knowing that Juana wants to go into a specialty where work/life balance is challenging, the attending could have offered to talk with her about how to create balance between career and personal/family life.*

- 1. **Bystander (ARISE Approach):** Whether there were bystanders or not in this scenario consider the role of a bystander or bystanders. How could they address this situation? Should it be done now or later?

What could be done to support or protect the recipient?

*Dr. Hakim, as the bystander, could have addressed the attending’s microaggressions at the time they were said or afterwards, in private. Given the power differentials, it would be best to address the comments later in private, if at all. If Dr. Hakim has a good relationship with Dr. Hernandez, he could have said, “I know you were trying to warn Juana about how difficult it can be to balance life as a surgeon with family, but she seemed offended by the comment. I’m telling you this because I know you meant well, but you seemed to assume that she would want family, and that if she were to have a family, that she would be the primary caretaker of her children and have to make difficult decisions between work and career. I know you to be a person who values equity and inclusion, so I was pretty surprised to hear you make those comments. You have never spoken to me about work/life balance, or whether I would have to make difficult choices between my career and family, and I wonder if this is because I am male? I know you care a great deal about your students, so I wanted to talk with you about this because I would hate for you to accidentally say something like this again without realizing how it came across.”*

What could be said to the source that could help them become aware of the microaggression while keeping them engaged in the interaction? What are ways to help the source re-establish their relationship with the recipient (s) and restore or protect their reputation? *Dr. Hakim could also be an active bystander by checking in on Juana after the interaction to see how she is doing and to offer validation and support.*

1. *Sexual Harassment of Women: Climate, Culture and Consequences in Academic Sciences, Engineering and Medicine.* Washington DC: National Academies of Sciences, Engineering and Medicine;2018.

2. Tsugawa Y, Jena A, Figueroa J, Oray E, Blumenthal D, Jha A. Comparison of Hospital Mortality and Readmission Rates for Medicare Patients Treated by Male vs Female Physicians *JAMA Internal Medicine.* 2017;177(2):206-213.

3. Sarsons H. Interpreting signals in the labor market: Evidence from medical referrals In:2017.

4. Morgan H, Purkiss J, Porter A, et al. Student evaluation of faculty physicians: Gender differences in teaching evaluations. *Journal of Women’s Health* 2016;25(5):453-456.

5. Files J, Mayer A, Ko M, et al. Speaker Introductions at Internal Medicine Grand Rounds: Forms of Address Reveal Gender Bias. *Journal of Women’s Health* 2017;26(5):413-419.

6. Reed D, Enders F, Lindor R, McClees M, Keith D. Gender differences in acafdemic productivity and leadership appointments of physicians throughout academic careers. *Academic Medicine* 2011;86(1):43-47.

7. Jolly S, Griffith K, DeCastro R, Stewart A, Ubel P, Jagsi R. Gender differences in time spent on parenting and domestice responsibilities by high-achieving young physician-researchers *Annals of Internal Medicine.* 2014;160(5):344-353.

8. Guille C, Frank E, Zhao Z. Work-Family Conflict and the Sex Difference in Depression Among Training Physicians *JAMA Internal Medicine.* 2017;177(12):1766-1772.

9. Paturel A. Why women leave medicine. *AAMC.* 2019. <https://www.aamc.org/news-insights/why-women-leave-medicine>.

10. Cheung F, Ganote C, Souza T. *Microaggressions and microresistance: Supporting and empowering students* 2016.

**Case 6: Advised Away**

Isabella is a Mexican-American third-year medical student, who is meeting her advisor in a group advising meeting to discuss her specialty choice and upcoming applications to residency programs. Her grades and Step 1 scores are very strong, and she has been very active in extracurricular service-based clinical activities with the Latinx community, such as leading the school’s Spanish-speaking student outreach clinic. She has developed an interest in orthopedic surgery and excitedly tells the group and advisor about her intent to apply to this specialty. Upon hearing this, the advisor shakes his head and states, “No, no, no! You can’t go into Ortho! You need to go into primary care. Your people need you!”

**Discussion Guide**

**Case 6: Advised Away**

1. Who was the recipient of the microaggression? What aspect of the recipient’s identity was the target of the microaggression?

*In this case, Isabella was the recipient of the microaggression from the advisor, and the microaggression was based on her Mexican-American ethnicity.*

2. Who was the source of the microaggression? What is the direction of the hierarchy in the relationship between the recipients and the source? For example, was the source in a position of authority, or was their position lateral or subordinate to the recipient?

*The source was the advisor, who is in a position of power and authority over Isabella.*

3. What was the nature of the microaggression? How it could have hurtful impact on the recipient?

*The advisor seemed to assume that Mexican-American students such as Isabella should stick to primary care specialties so they can better serve “their people.” This could have a hurtful impact on Isabella, as it could create a sense of obligation for her to go into a primary care specialty she has little interest in. It could promote a false division between the “advisor’s people” and “her people,” and/or could reinforce a stereotype that it is the duty of physicians who are underrepresented in medicine to work with underserved populations.*

4. What is the historical, structural, cultural context of the microaggression?

*Key Point*

*a. The concept of the diversity tax (also known as the minority tax) is important in this context. The diversity tax refers to the expectation that people of color take on the burden of addressing and solving the diversity issues for their organization^1^ or communities. It is a tax because individuals of color are often not compensated for even recognized for this extra work.^1^*

5. How might the recipient and source be viewing the situation differently?

[This section is critically important in the triangle model. Do not try to establish who is right or wrong, or what should have been said or done. Instead, focus on each person involved and, given that people are complex and not all good or all bad, try to understand what their world view may be. This helps you make an informed decision about how to proceed].

*The source likely thought that they were being helpful, in advising Isabella to work with patients from a culture she has already worked with in the Spanish-speaking student outreach clinic, or perhaps given her personal background of being a Latina. She may have been aware of the literature suggesting that matching the race between a patient and a physician can result in better clinical outcomes. However, Isabella is likely viewing this situation as a microaggression. The advisor’s comment suggests that it is an obligation for students who are underrepresented in medicine to go into primary care and to focus their careers on treating underserved populations.*

| **Recipient-ACTION Approach^2^** | **Source-ASSIST Approach** | **Bystander-ARISE Approach** |
| --- | --- | --- |
| **A**sk a clarifying question | **A**cknowledge your bias | **A**wareness of microaggression |
| **C**ome from curiosity | **S**eek feedback | **R**espond with empathy (avoid judgment) |
| **T**ell what you observed | **S**ay you are sorry | **I**nquiry of facts |
| **I**mpact Exploration | **I**mpact, not Intent | **S**tatements that start with ‘I” |
| **O**wn thoughts and feelings | **S**ay **T**hank You | **E**ducate and **E**ngage |
| **N**ext steps |  |  |

1. Discuss responses from each member involved in the interaction that could repair and re-establish relationships and restore reputations

**a. Recipient (ACTION Approach):** Given the circumstance what are ways the recipient could address this (including not addressing it in the moment)?

*Isabella could choose not to address the advisor’s comments in that moment, and she could address the advisor later, in private. Alternatively, she could choose not to address the comments at all, as this is not her responsibility. Should she choose to address the advisor’s comments, she could ask the advisor what he meant by his comment. She could also say something along the lines of, “My people are not only patients of Hispanic descent, but all patients, and I can serve all patients well by going into Orthopedics. I want to be in a field that I am passionate about, and Ortho is where I want to be.”*

**b. Source (ASSIST Approach):** Put yourself in the role of the source. Imagine that you become aware of your microaggression. What could you say or do to repair and re-establish relationship your relationship with the recipient (s)?

*The advisor could have used mindfulness and present-moment awareness skills to notice the reaction on the faces of Isabella and the other students, and use values clarification to realize the discrepancy between their intention of helping Isabella and the outcome of offending her. Also, they should have focused on the impact of the comment rather than the intention of helping Isabella. A simple comment such as, “Oops!” could convey that they realized they made a mistake. They could have then taken responsibility for their assumptions, apologized for the comment, and expressed a commitment to change the behavior in the future. Knowing that Isabella wants to go into a highly competitive specialty, the advisor could have offered to meet with her in additional office hours to help her with her application.*

**c. Bystander (ARISE Approach):** Whether there were bystanders or not in this scenario consider the role of a bystander or bystanders. How could they address this situation? Should it be done now or later?

- What could be done to support or protect the recipient?
- What could be said to the source that could help them become aware of the microaggression while keeping them engaged in the interaction? What are ways to help the source re-establish their relationship with the recipient (s) and restore or protect their reputation?

*All of the other students in the group were bystanders in this scenario, and they could have intervened in a number of ways. First, one of them could have addressed the advisor in the moment, with a simple statement like “Ouch!,” letting the advisor know that something offensive was said. If the advisor didn’t act on that clue, the bystander could have gone on to say something along the lines of, “I know you meant well, but your statement to Isabella assumes that people of color have an obligation to work with the underserved, and I don’t think this is fair.” They could have also said something like, “I think that we all share the duty to take care of underserved populations, and I hope that Isabella can go into whatever specialty she chooses.” Second, a bystander could have approached the advisor later, in private, to share their concern. Finally, a bystander could approach Isabella after this interaction to offer validation and support such as by asking hers how he is doing after the incident, texting her, or calling her.*

*After the interaction, a bystander such as another student in the advising group could approach the advisor, saying, “Professor, I have to tell you that I was surprised you told Isabella she has to go into primary care because her people need her. She seemed really hurt by your assumption that minority students have to care for underserved populations. I know you to be a person who values inclusion and equity, so it seemed strange that you were so adamant that she has to go into primary care and not ortho. And I wanted to tell you this because I know you are advising students because you really care about us, and I would hate for you to accidentally say something like this again without realizing how it came across.”*

1. Ackerman-Barger K, Boatright D, Gonzalez-Colaso R, Orozco R, Latimore D. Seeking Inclusion Excellence: Understanding Racial Microaggressions as Experienced by Underrepresented Medical and Nursing Students. *Academic Medicine.* 2020;95(5):758-763.

2. Cheung F, Ganote C, Souza T. *Microaggressions and microresistance: Supporting and empowering students* 2016.

**Case 7: A Hidden Curriculum**

During an interview, a health professions student shared the following experience from one of their didactic classes:

“Our class went through this whole case study to review for a licensing exam. The case said,

*‘you have a patient who practices Islam and you need to figure out their nutritional needs and figure out some of their diet as a part of their care plan.’*

There were certain questions related to the case that we had to answer, one of which was *‘How do you make sure that you are being sensitive to the patient’s religion?’* While leading the class discussion the professor said, ‘It is important to make sure that if the patient is a woman that you talk to both her and her husband because that’s the way that Islam works’.”

**Discussion Guide**

**Case: 7 Hidden Curriculum**

1. Who was the recipient of the microaggression? What aspect of the recipient’s identity was the target of the microaggression?

*In this scenario there is no clear recipient of the microaggression. However, there may be students in the class who practice Islam and feel targeted by the statement.*

2. Who was the source? What is the direction of the hierarchy in the relationship between the recipients and the source? For example, was the source in a position of authority, or was their position lateral or subordinate to the recipient?

*In this case the source of the microaggression was the professor who is in a position of power over the students by evaluating them and making key decision about their academic success.*

3. What was the nature of the microaggression? How it could have hurtful impact on the recipient?

*This microaggression was built into the curriculum, a concept called “hidden curriculum” where implicit curriculum is expressed that represents attitudes, biases, and behaviors which are conveyed indirectly without aware intent ^1^. The professor made an assumption about the provider-patient relationship based on the religion of the patient. Because the patient was a female, the professor assumed that her husband needed to be an equal participant in the conversation around her care. The case also did not specify that the patient was female or there with her husband indicating that gender was not the intended focus of the question. The professor bringing it up now reinforces a stereotype and also suggests heteronormative perspective that if she is married it is to a man.*

*While this statement was not directed at a specific person it is still hurtful to students in the classroom who are Muslim. The statement is also harmful to the class and overall learning community because it propagates a stereotype. Working in health professions means making associations. If students learn to automatically connect a religion to an (incorrect) way of practice, then it is likely that they will replicate this practice with each patient they see with that identity. The statement could give students the idea that every person who practices Islam believes that husbands must be involved in all aspects of their wives lives and that the husband is to be given equal importance in discussions about a woman’s health.*

4. What is the historical, structural, cultural context of the microaggression?

*Key Points*

- 1. *The word choice in the professor’s statement, “That’s the way it works” greatly oversimplifies a religion had over 1.5 billion observers in 2009. ^2^ Religious practices are not necessarily the same as cultural practices. A Muslim person may have varying levels of religious practice and have many identities (gender identity, sexual orientation, race/ethnicity etc.) which influence the customs they follow. This comment makes it seem that because someone is Muslim they therefore must follow a certain custom or type of behavior.*
  2. *Islamophobia is a fear-based prejudice and discrimination against Islam or people who practice Islam ^3^, while prevalent in history for centuries, has become more rampant over the last few decades. ^4,5^ CAIR, the Council on American-Islamic Relations, reported a total of 10,015 anti-Muslim bias incidents between 2014 and 2019, with some of the most frequent being harassment, hate crimes, bullying and denial of religious accommodations. 69% of Muslim women who wear a hijab have reported experiencing discrimination at least once. ^6^ The current political situation, particularly the ban on foreign nationals entering the US from seven predominantly Muslim countries and the prolonged court battles to uphold new versions of the executive order ^7^, caused a significant increase in the number reports of anti-Muslim incidents. ^4^ Anti-Muslim bias can manifest in many ways and venues, such as in the media and interpersonal interactions.*
  3. *Healthcare is not immune to these anti-Muslim biases and stereotypes. A content analysis of articles in MEDLINE found latent themes that Muslims are negatively affected by tradition and are against biomedical healthcare delivery, implying that being an observant Muslim is a health risk. ^8^*
  4. *Stereotyping and hate crimes against Muslim women are more prominent than for men ^6^. Mahr describes these stereotypes as “depicting them as a homogenous group supporting regressive beliefs, values, and practices incompatible with modern life. This completely disregarding the history and diversity of Muslim women”. The microaggression discussed in this case builds upon stereotyped assumptions about the gender dynamics within a Muslim family.*

5. How might the recipient and source be viewing the situation differently? [This section is critically important in the triangle model. Do not try to establish who is right or wrong, or what should have been said or done. Instead, focus on each person involved and, given that people are complex and not all good or all bad, try to understand what their world view may be. This helps you make an informed decision about how to proceed]

*The professor may have thought that they were being culturally competent by tailoring their approach with the patient to the patient’s background and teaching these skills to the students. They may also be strictly following the curriculum and did not think to make any changes to the written approach. However, the students in the room may feel attacked and/or hurt by the statement.*

| **Recipient-ACTION Approach^9^** | **Source-ASSIST Approach** | **Bystander-ARISE Approach** |
| --- | --- | --- |
| **A**sk a clarifying question | **A**cknowledge your bias | **A**wareness of microaggressio |
| **C**ome from curiosity | **S**eek feedback | **R**espond with empathy) avoid judgment) |
| **T**ell what you observed | **S**ay you are sorry | **I**nquiry of facts |
| **I**mpact Exploration | **I**mpact, not Intent | **S**tatements that start with ‘I” |
| **O**wn thoughts and feelings | **S**ay **T**hank You | **E**ducate and **E**ngage |
| **N**ext steps |  |  |

6. Discuss responses from each member involved in the interaction that could repair and re-establish relationships and restore reputations

**a. Recipient (ACTION Approach):** Given the circumstance what are ways the recipient could address this (including not addressing it in the moment)? What can the recipient do to maintain and restore their well-being after this microaggression?

*While there is no direct recipient in this situation a student may have taken some of the following approaches:*

- *It should be noted that the student-recipient does own the responsibility of addressing a microaggression. If the student chooses to, they may address the situation in the moment or in private after. However, because of the power dynamic between student and professor, the student should consider addressing the situation in private.*
- *The student could begin a discussion with curiosity by asking the professor a clarifying question, such as “Do you mean to say that we should treat all Muslim women, or all women, in this manner?”*
- *The student may narrate what they observed and/or share their own thoughts and feelings about the statement by saying “Your answer to the question seemed to state that because the patient was female and Muslim that we as providers should discuss her private health information with both her and her husband. While I understand that you were trying to be culturally appropriate, the suggestion that all Muslim women should be treated this way because of their religion and gender seems like a broad assumption. That made me feel uncomfortable”.*

**b. Source (ASSIST Approach):** Put yourself in the role of the source. Imagine that you become aware of your microaggression. What could you say or do to repair and re-establish relationship your relationship with the recipient (s)?

*If the professor observed the faces of the students or their reactions, he/she might have realized the impact the statement had on them. When the professor realized that the statement perpetuated a stereotype and made a broad assumption, they could use a simple statement such as “Oops!” to indicate that they made a mistake. The professor could then continue by acknowledging the microaggression and the impact of the statement by apologizing. The professor may use language such as “I recognize that in this instance I made an assumption about a patient and her wishes based on her religion. It was incorrect of me to make this broad assumption and perpetuate a stereotype about gender roles in this way. I understand that this is hurtful statement and I apologize. I would welcome your feedback on how I can better discuss cultural practices and preferences in our class. ”*

*If a student discusses the situation with the professor in the moment or after the professor should listen carefully to what the student has to say, thank the student(s) for addressing the situation with them, and acknowledge the mistake and impact of the statement.*

**c. Bystander (ARISE Approach):** Whether there were bystanders or not in this scenario consider the role of a bystander or bystanders. How could they address this situation? Should it be done now or later?

- What could be done to support or protect the recipient?
- What could be said to the source that could help them become aware of the microaggression while keeping them engaged in the interaction? What are ways to help the source re-establish their relationship with the recipient (s) and restore or protect their reputation?

*The bystanders could address the situation in the moment or in private after the class had ended.*

***Bystander to Professor***

*In the moment, a bystander could have responded with a short statement such as “Ouch!” to show the professor that the statement was hurtful or in an inquisitive manner by saying something such as “Did you mean to say that we should treat all people with a certain identity in the same way and that Muslim men must be included in all private conversations about their wife’s health?”*

*The bystander could also use I statements to educate and engage with the professor. An example of this approach could be “I understand that you were trying to model for us how to take cultural practices and preferences into account when we engage with patients. This is an important skill. However, in this case, your comment may have suggested that we should treat all Muslim women in this manner. This is a broad assumption that all people in a religious group have the same preferences. It also perpetuates a stereotype about gender roles in general and within the Muslim religion. I’m sharing this with you because I know that you care about our ability to engage with patients and that you would not want to say something like this again without realizing how it may come across”. This longer debrief may be more productive in a private setting after the class.*

***Bystander to Peer***

*The bystander could also use observations and reflection skills to see if any of their classmates appear upset by the statement. If the bystander has a preexisting relationship with that person they could check in with them after class by saying something such as “I noticed that when the professor was discussing the answers to the case that you looked uncomfortable or upset. How are you doing? Is there anything that I can do to support you?”*

1. Alsubaie MA. Hidden Curriculum as One of Current Issue of Curriculum. *Journal of Education and Practice* 2015;6(33):125-128.

2. *Mapping the Global Muslim Population* Pew Research Center 2009.

3. Merriam-Webster. In:2020.

4. CAIR. *The Bias Brief: Trump’s Impact on Anti-Muslim Bias* 2019.

5. Buikstra E, Eley RM, Hindmarsh N. Informing rural and remote students about careers in health: factors influencing career decisions. *Aust J Rural Health.* 2007;15(5):289-295.

6. Mahr F, Nadeem T. Muslim Women and Islamophobia. In: Moffic HS, Peteet J, Zakaria A, Awaad R, eds. *Islamophobia and Psychiatry* Springer 2019.

7. ACLU. Timeline of the Muslim Ban <https://www.aclu-wa.org/pages/timeline-muslim-ban>. Published 2020. Accessed.

8. Laird LD, Marrais Jd, Barnes L. Protraying Islam and Muslims in MEDLINE: a content analysis *Social Science and Medicine.* 2008;65(12):2425-2439.

9. Cheung F, Ganote C, Souza T. *Microaggressions and microresistance: Supporting and empowering students* 2016.
