# Supplementary material for: Addressing Microaggressions in Academic Health: A Workshop for Inclusive Excellence
Source: MedEdPORTAL. 2021 Feb 11;17:11103. doi: 10.15766/mep_2374-8265.11103 (PMC7880252; doi:10.15766/mep_2374-8265.11103)
Supplement: Supplementary file 1 — Cases & Facilitator Guides.docxPowerPoint.pptxTimetable for Learning Activities.docxHandouts for Learners.docxCore Definitions.docxPre- & Posttest.docx [file mep_2374-8265.11103-s001.zip › B. PowerPoint.pptx]

## Slide 1
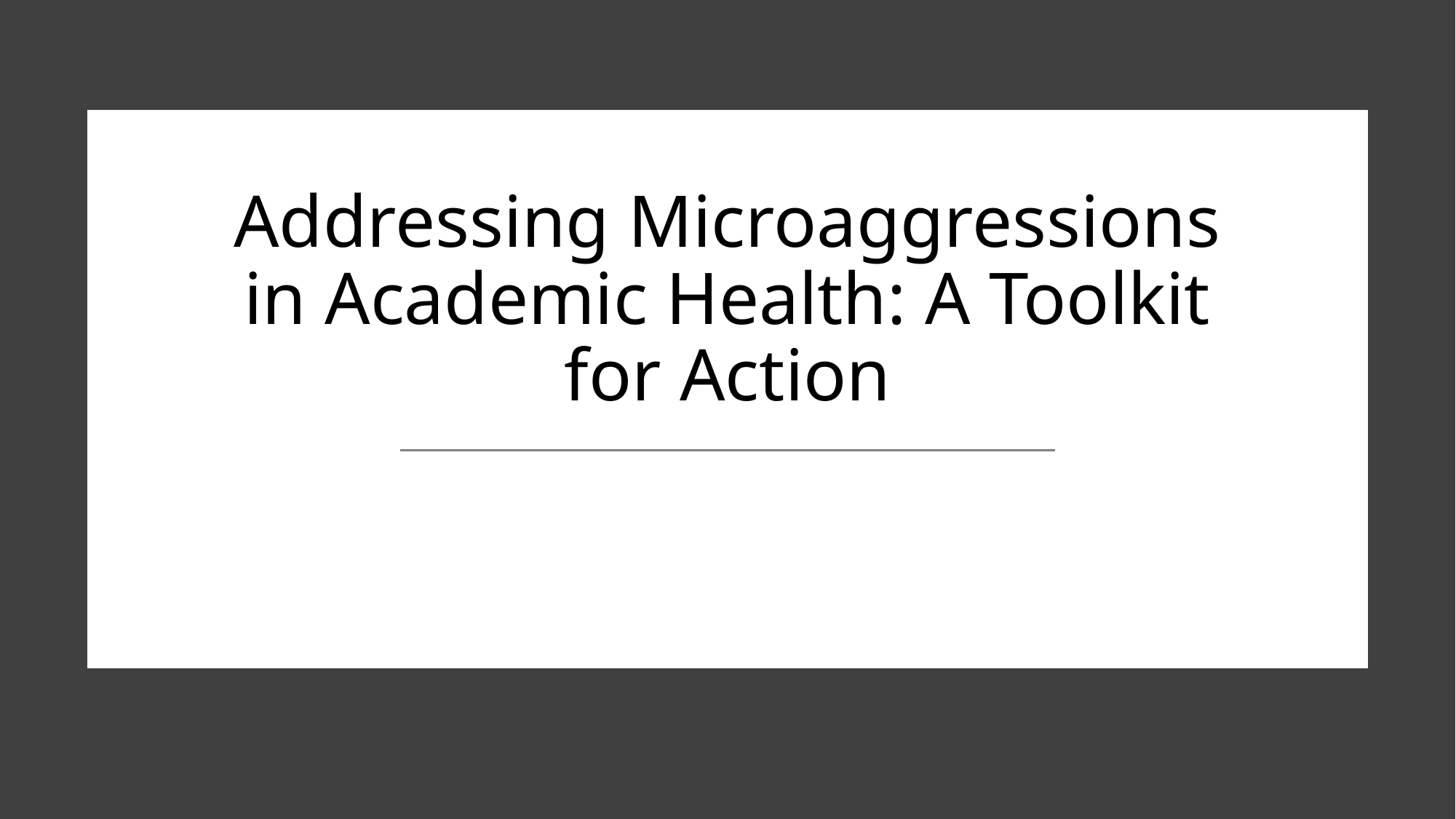

# Addressing Microaggressions in Academic Health: A Toolkit for Action

## Slide 2
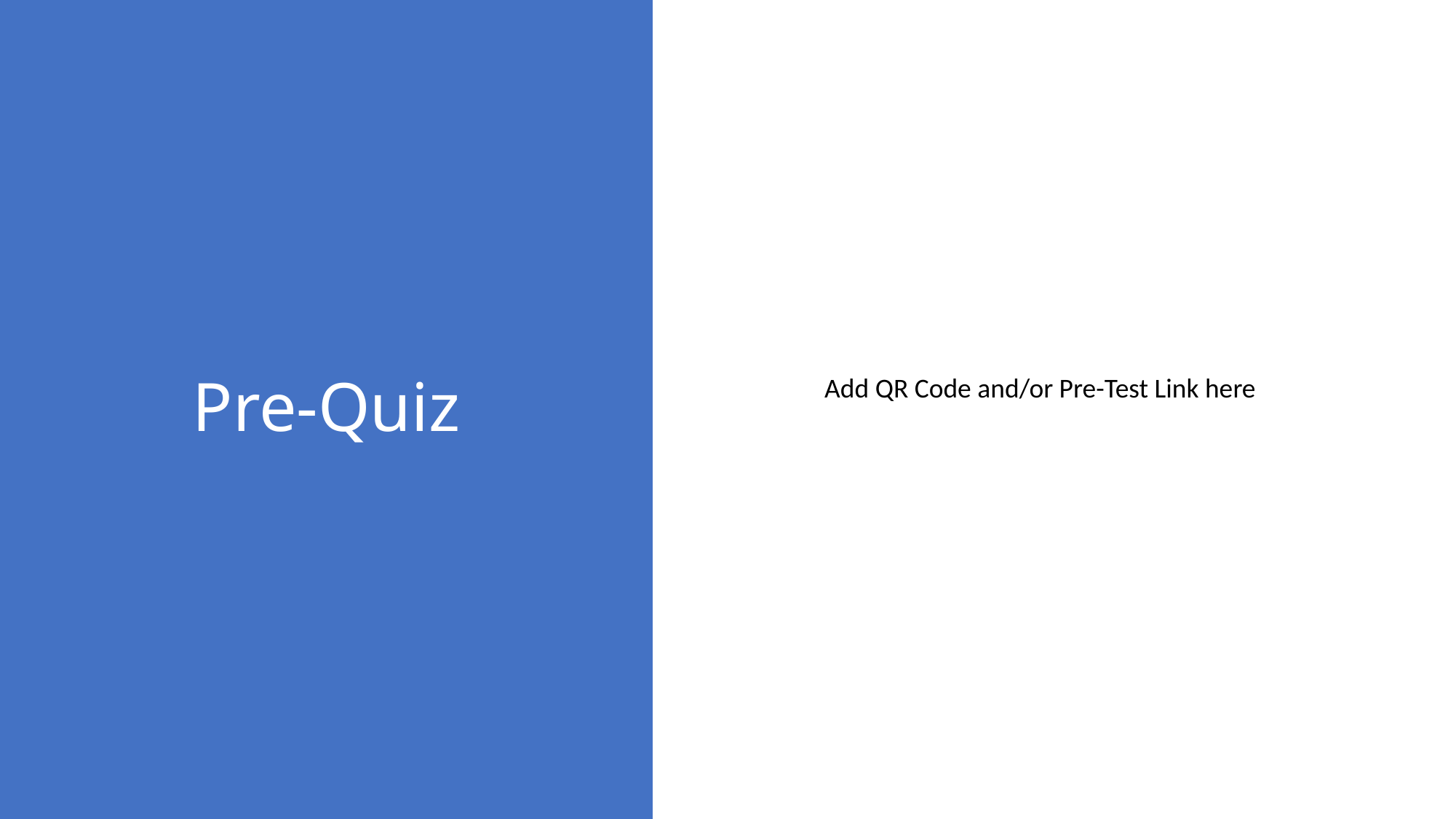

# Pre-Quiz
Add QR Code and/or Pre-Test Link here

## Slide 3
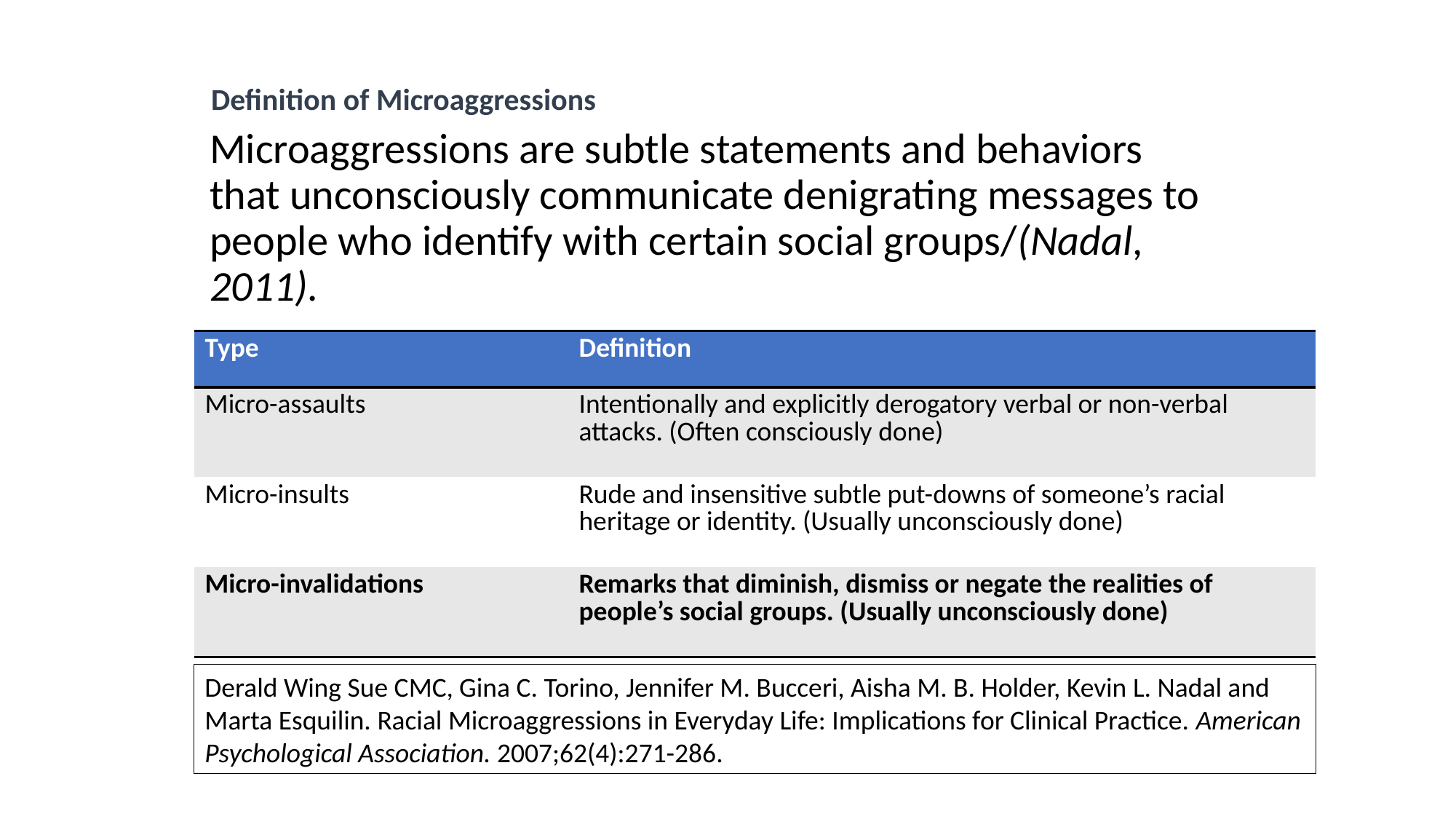

# Definition of Microaggressions
Microaggressions are subtle statements and behaviors that unconsciously communicate denigrating messages to people who identify with certain social groups/(Nadal, 2011).
| Type | Definition |
| --- | --- |
| Micro-assaults | Intentionally and explicitly derogatory verbal or non-verbal attacks. (Often consciously done) |
| Micro-insults | Rude and insensitive subtle put-downs of someone’s racial heritage or identity. (Usually unconsciously done) |
| Micro-invalidations | Remarks that diminish, dismiss or negate the realities of people’s social groups. (Usually unconsciously done) |
Derald Wing Sue CMC, Gina C. Torino, Jennifer M. Bucceri, Aisha M. B. Holder, Kevin L. Nadal and Marta Esquilin. Racial Microaggressions in Everyday Life: Implications for Clinical Practice. American Psychological Association. 2007;62(4):271-286.
Center for a Diverse Healthcare Workforce

## Slide 4
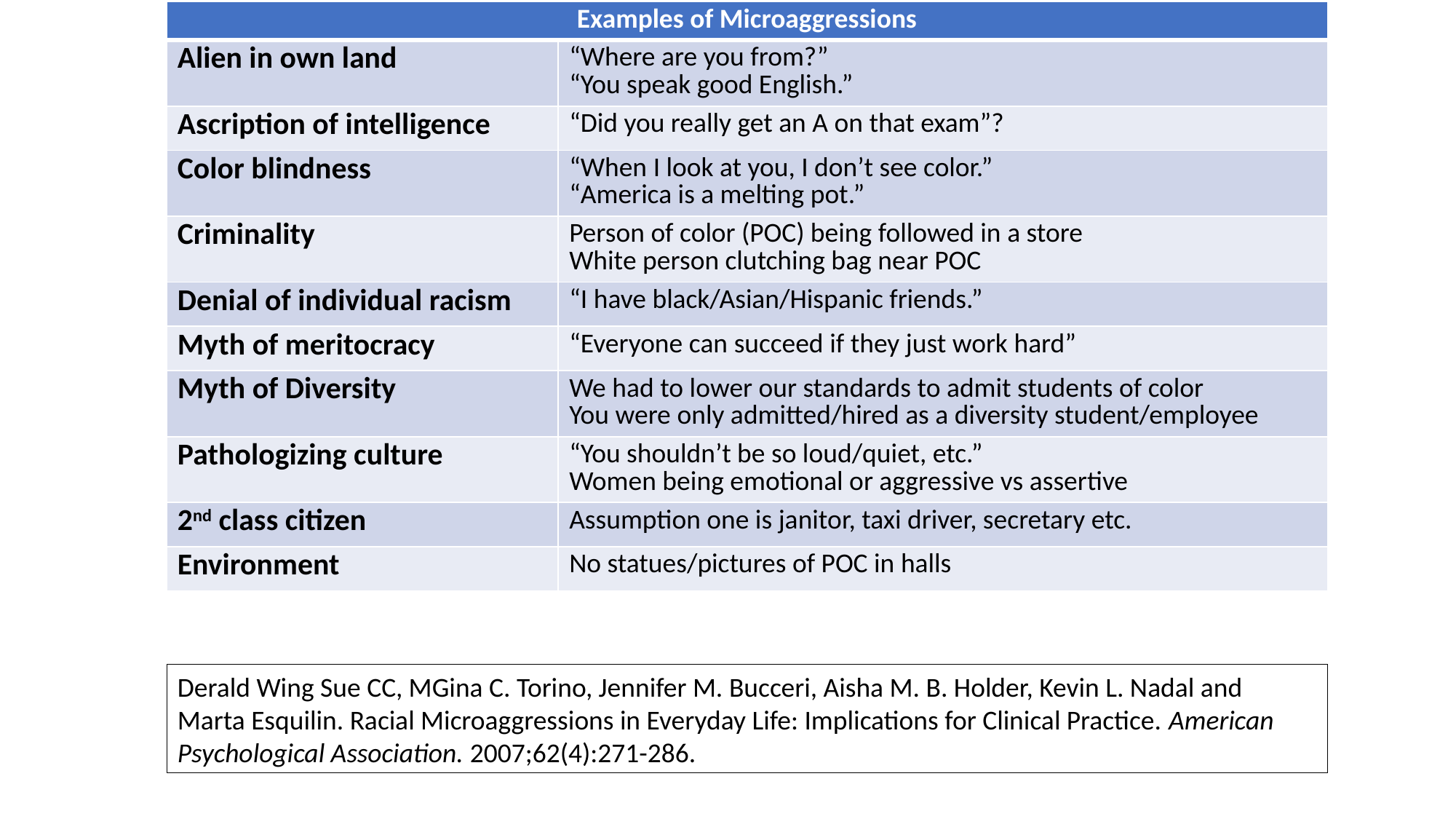

| Examples of Microaggressions | |
| --- | --- |
| Alien in own land | “Where are you from?” “You speak good English.” |
| Ascription of intelligence | “Did you really get an A on that exam”? |
| Color blindness | “When I look at you, I don’t see color.” “America is a melting pot.” |
| Criminality | Person of color (POC) being followed in a store White person clutching bag near POC |
| Denial of individual racism | “I have black/Asian/Hispanic friends.” |
| Myth of meritocracy | “Everyone can succeed if they just work hard” |
| Myth of Diversity | We had to lower our standards to admit students of color You were only admitted/hired as a diversity student/employee |
| Pathologizing culture | “You shouldn’t be so loud/quiet, etc.” Women being emotional or aggressive vs assertive |
| 2nd class citizen | Assumption one is janitor, taxi driver, secretary etc. |
| Environment | No statues/pictures of POC in halls |
# Microaggressions
Derald Wing Sue CC, MGina C. Torino, Jennifer M. Bucceri, Aisha M. B. Holder, Kevin L. Nadal and Marta Esquilin. Racial Microaggressions in Everyday Life: Implications for Clinical Practice. American Psychological Association. 2007;62(4):271-286.

## Slide 5
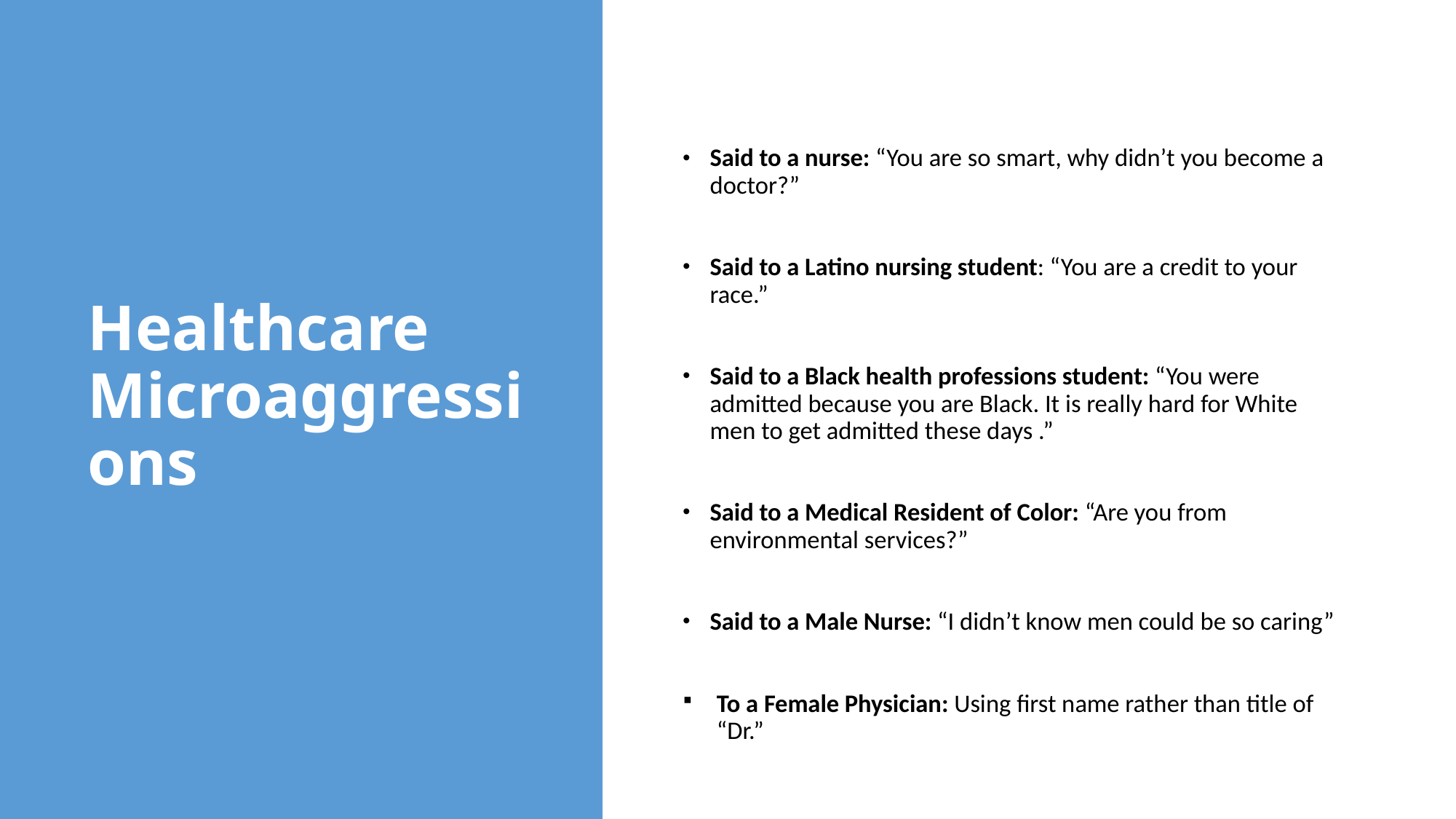

# Healthcare Microaggressions
Said to a nurse: “You are so smart, why didn’t you become a doctor?”
Said to a Latino nursing student: “You are a credit to your race.”
Said to a Black health professions student: “You were admitted because you are Black. It is really hard for White men to get admitted these days .”
Said to a Medical Resident of Color: “Are you from environmental services?”
Said to a Male Nurse: “I didn’t know men could be so caring”
To a Female Physician: Using first name rather than title of “Dr.”

## Slide 6
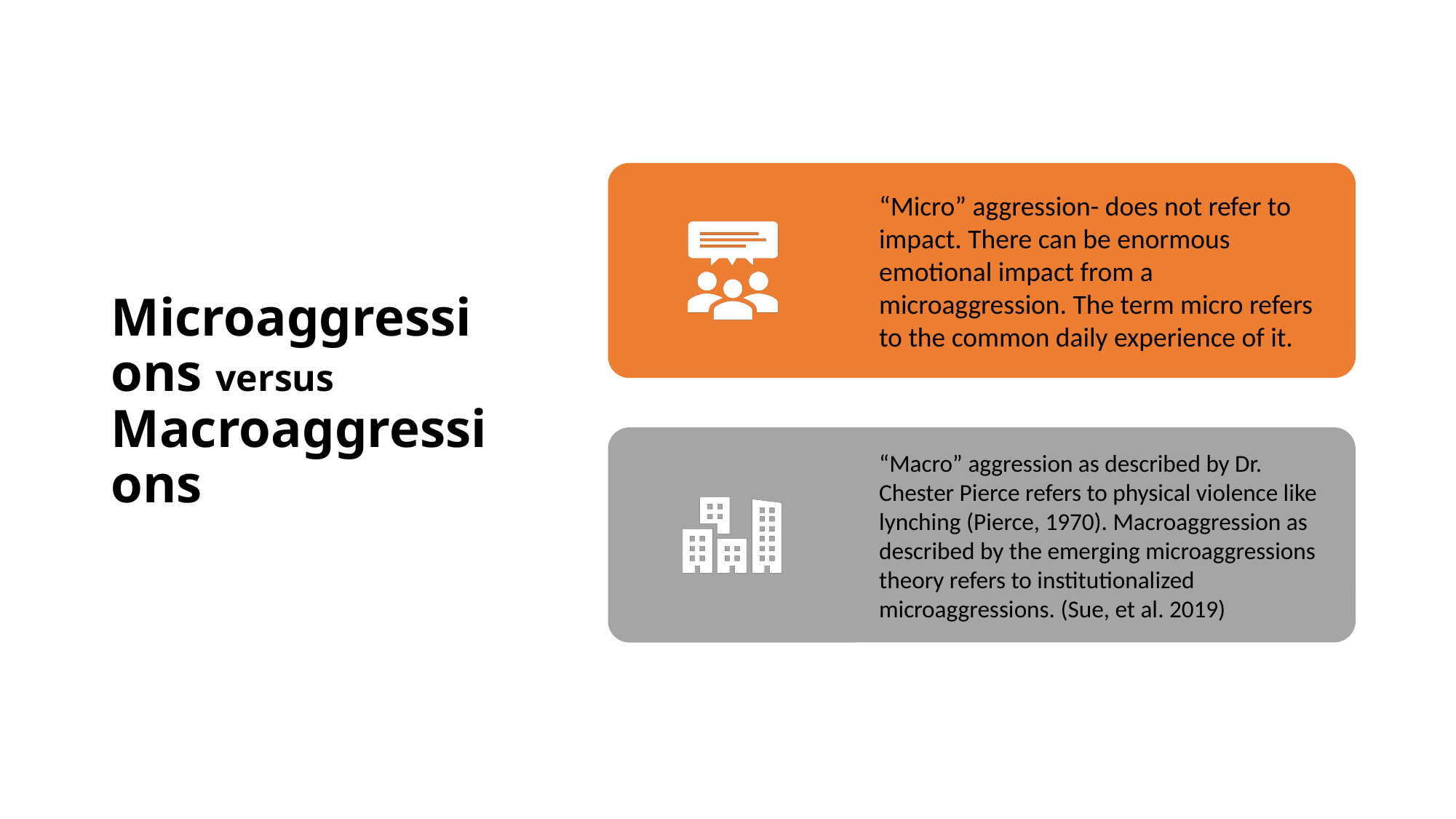

# Microaggressions versus Macroaggressions

## Slide 7
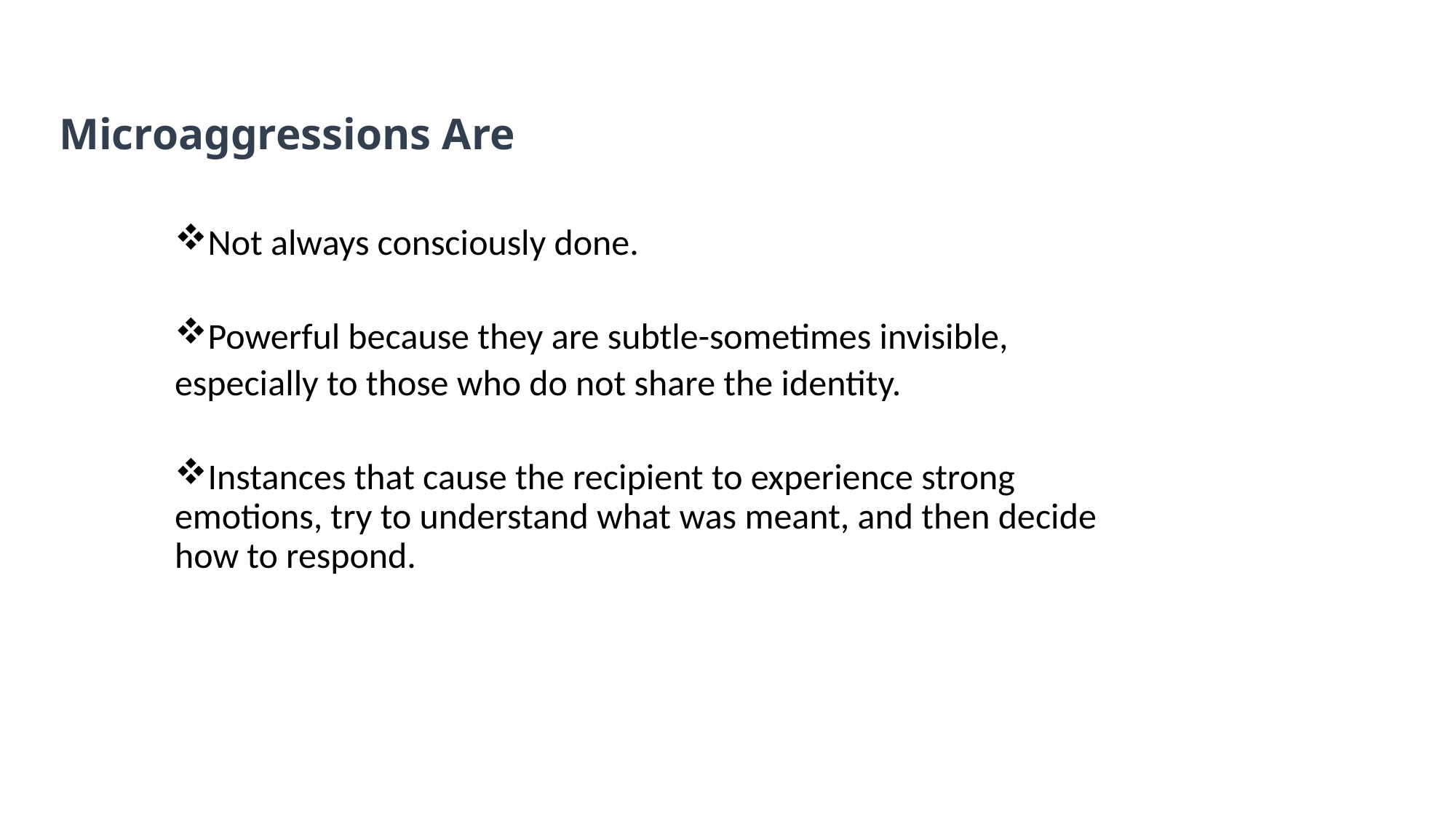

# Microaggressions Are
Not always consciously done.
Powerful because they are subtle-sometimes invisible,
especially to those who do not share the identity.
Instances that cause the recipient to experience strong emotions, try to understand what was meant, and then decide how to respond.

## Slide 8
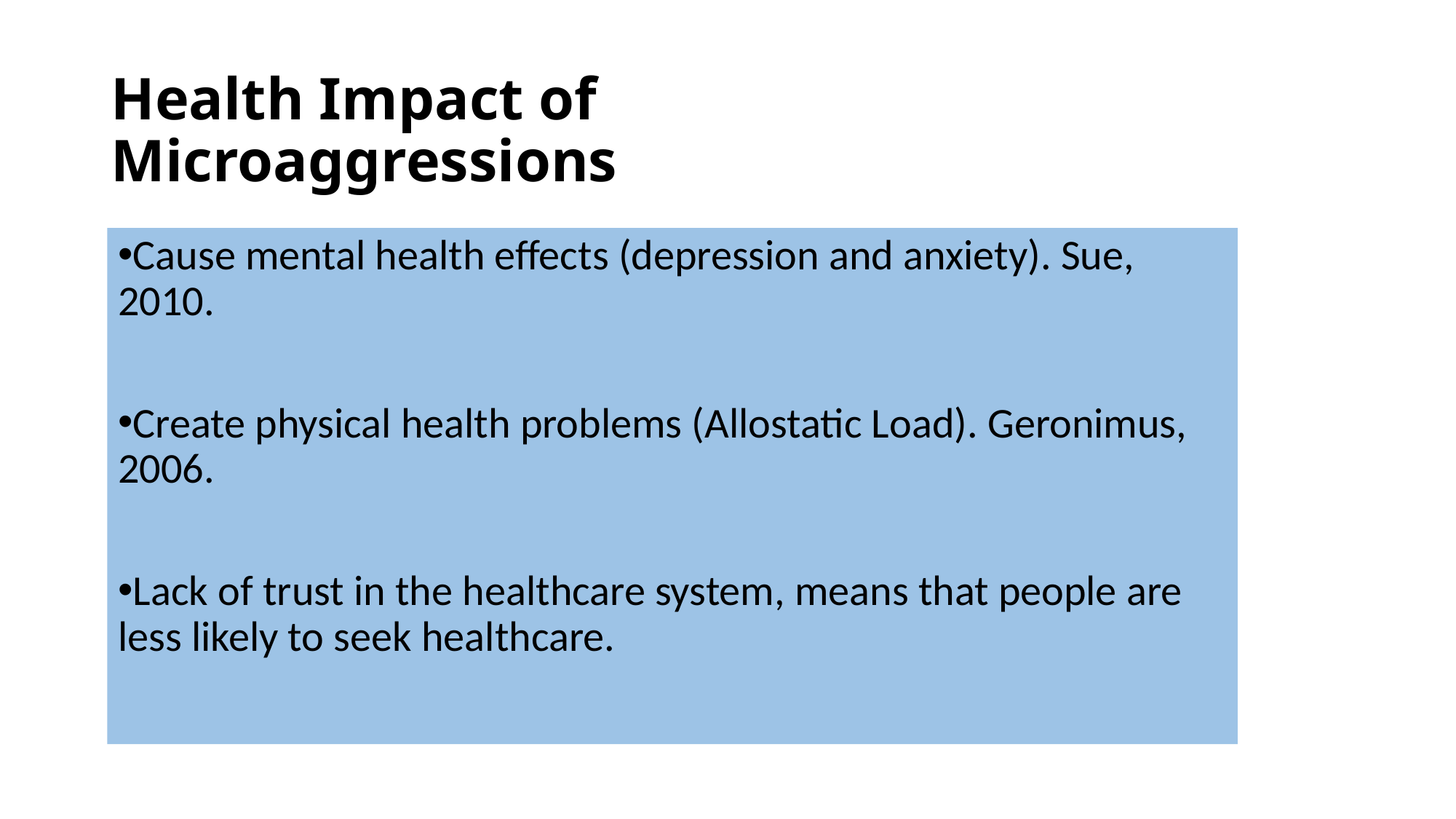

# Health Impact of Microaggressions
Cause mental health effects (depression and anxiety). Sue, 2010.
Create physical health problems (Allostatic Load). Geronimus, 2006.
Lack of trust in the healthcare system, means that people are less likely to seek healthcare.

## Slide 9
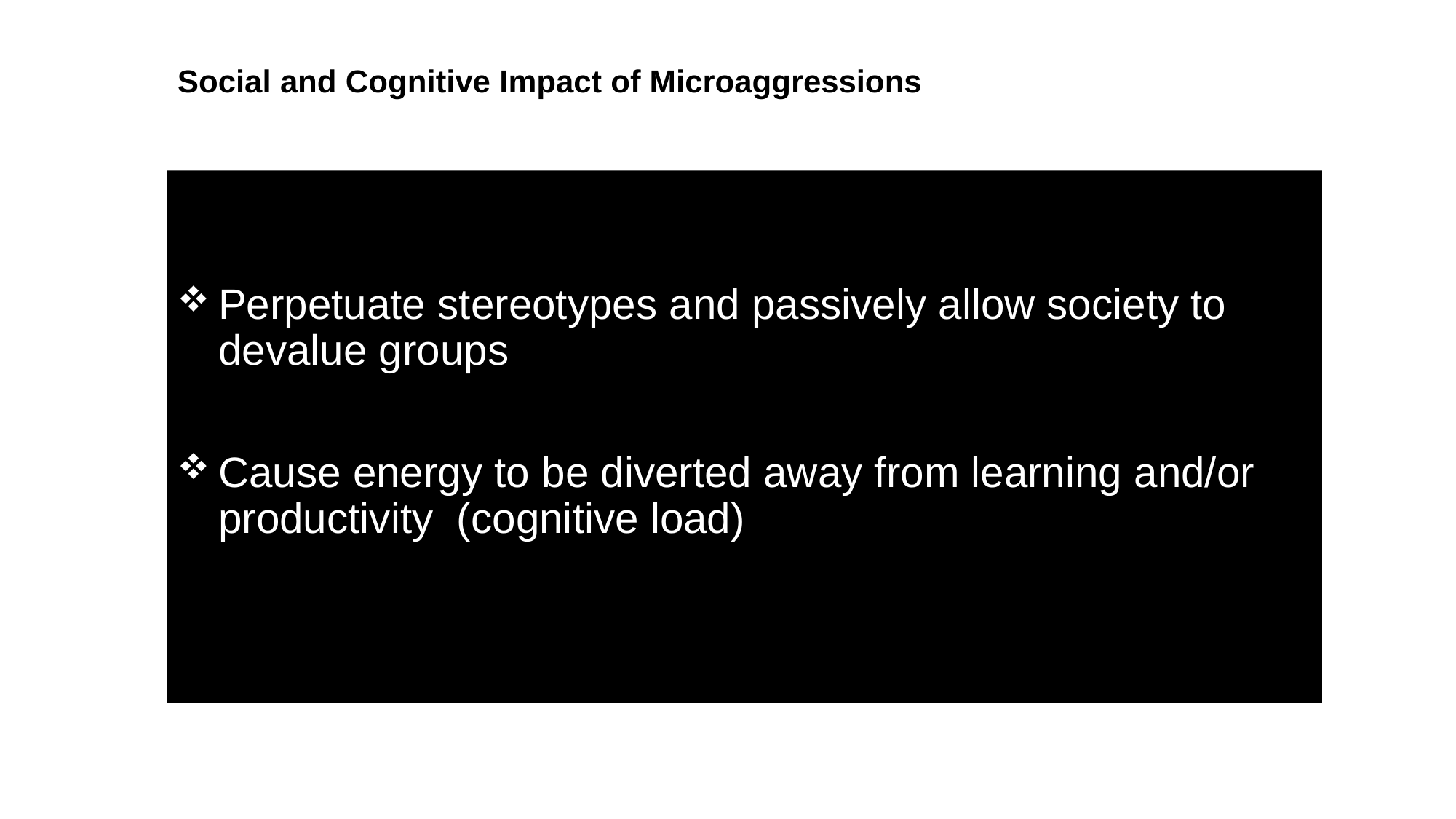

# Social and Cognitive Impact of Microaggressions
Perpetuate stereotypes and passively allow society to devalue groups
Cause energy to be diverted away from learning and/or productivity (cognitive load)
Center for a Diverse Healthcare Workforce

## Slide 10
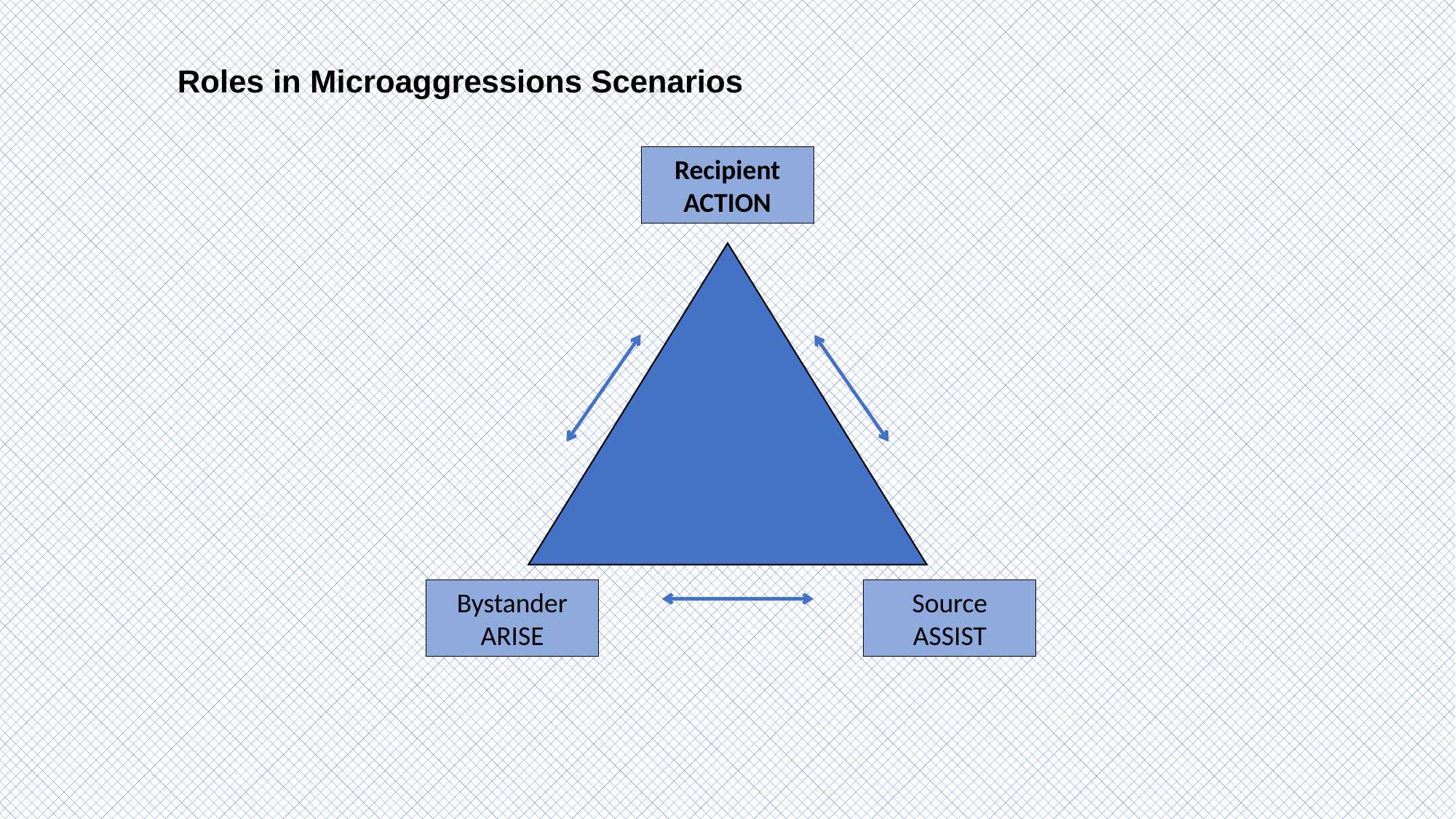

# Roles in Microaggressions Scenarios
Recipient
ACTION
Bystander
ARISE
Source
ASSIST

## Slide 11
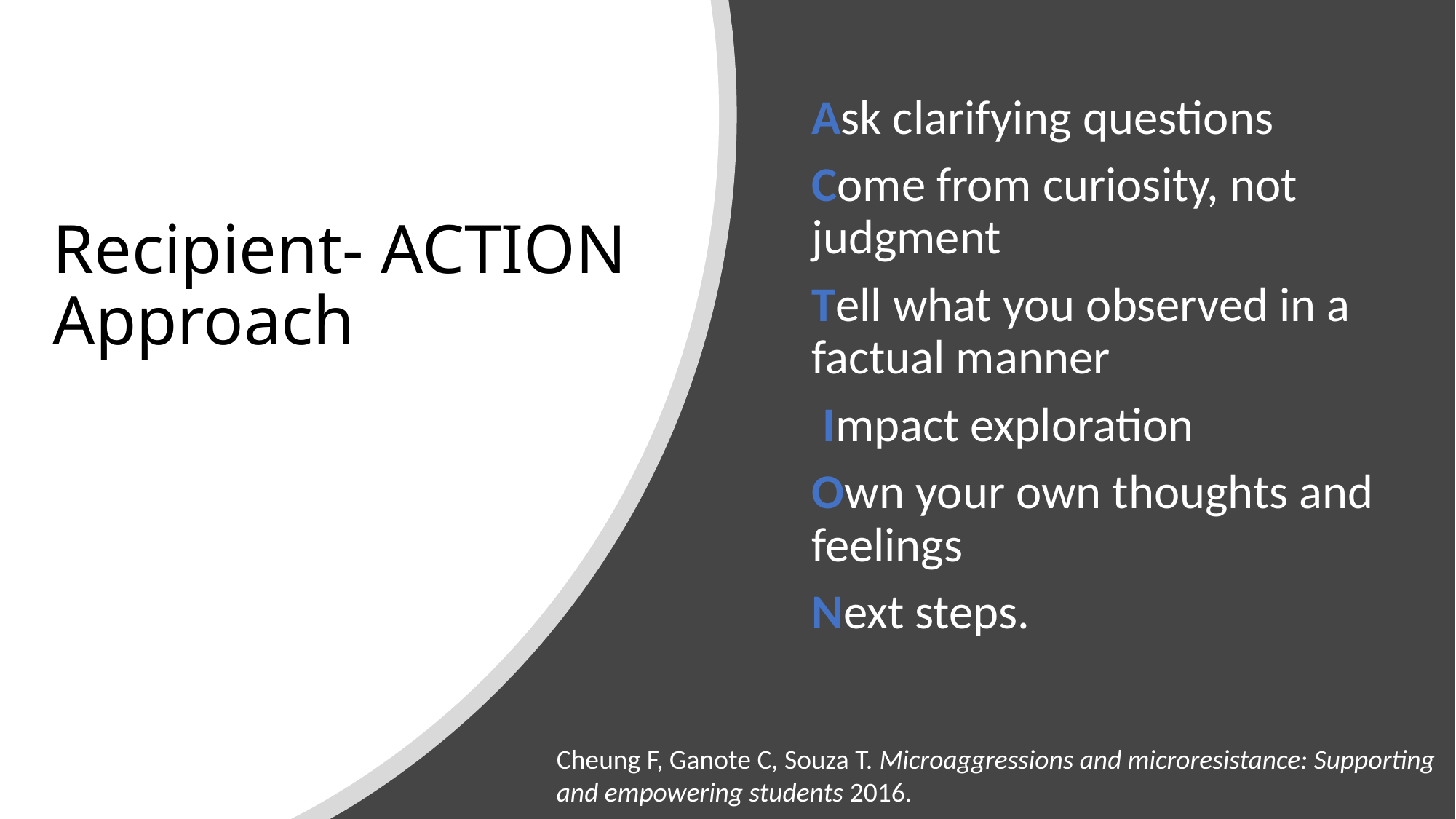

Ask clarifying questions
Come from curiosity, not judgment
Tell what you observed in a factual manner
 Impact exploration
Own your own thoughts and feelings
Next steps.
# Recipient- ACTION Approach
Cheung F, Ganote C, Souza T. Microaggressions and microresistance: Supporting and empowering students 2016.

## Slide 12
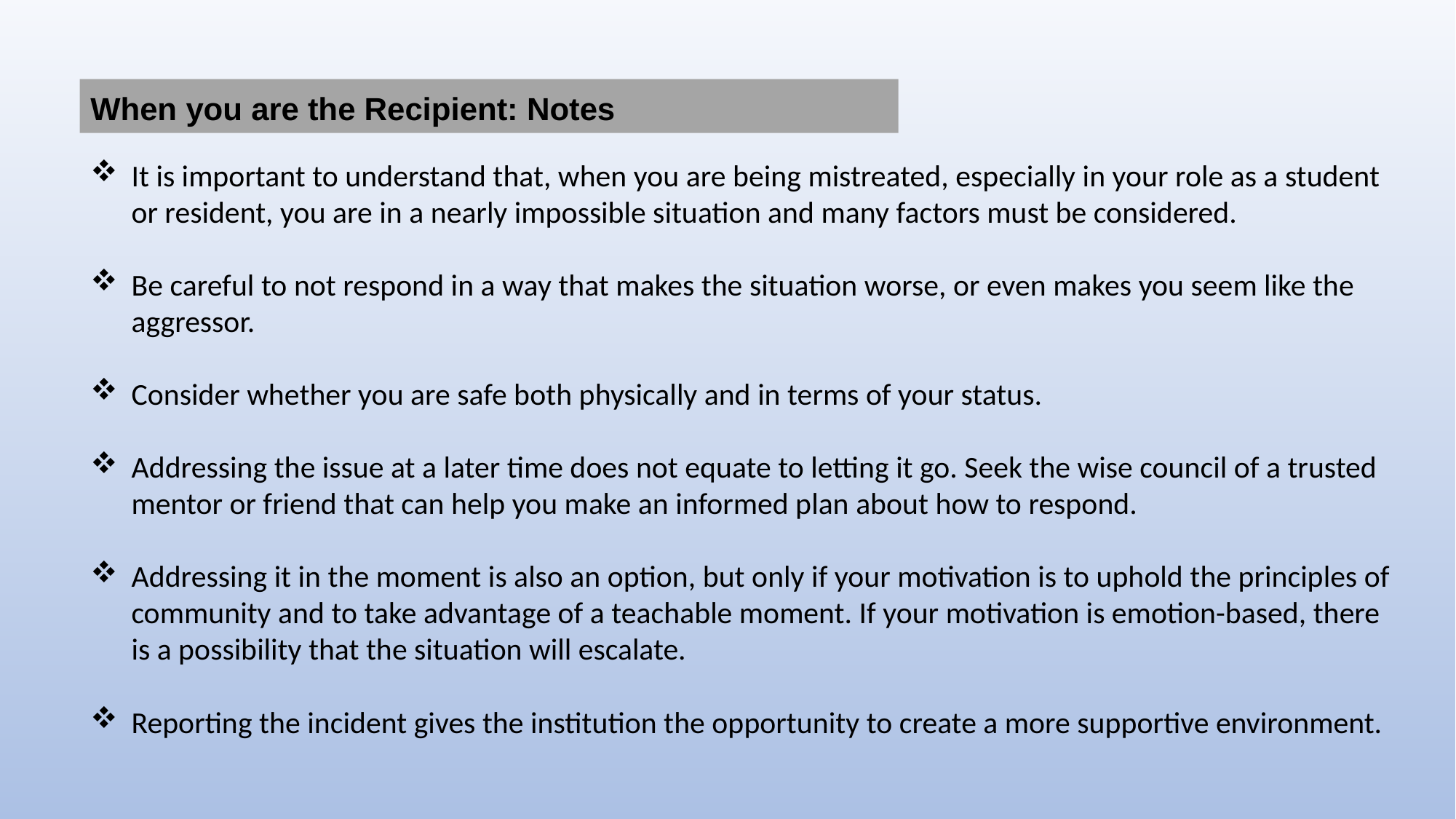

#
When you are the Recipient: Notes
It is important to understand that, when you are being mistreated, especially in your role as a student or resident, you are in a nearly impossible situation and many factors must be considered.
Be careful to not respond in a way that makes the situation worse, or even makes you seem like the aggressor.
Consider whether you are safe both physically and in terms of your status.
Addressing the issue at a later time does not equate to letting it go. Seek the wise council of a trusted mentor or friend that can help you make an informed plan about how to respond.
Addressing it in the moment is also an option, but only if your motivation is to uphold the principles of community and to take advantage of a teachable moment. If your motivation is emotion-based, there is a possibility that the situation will escalate.
Reporting the incident gives the institution the opportunity to create a more supportive environment.

## Slide 13
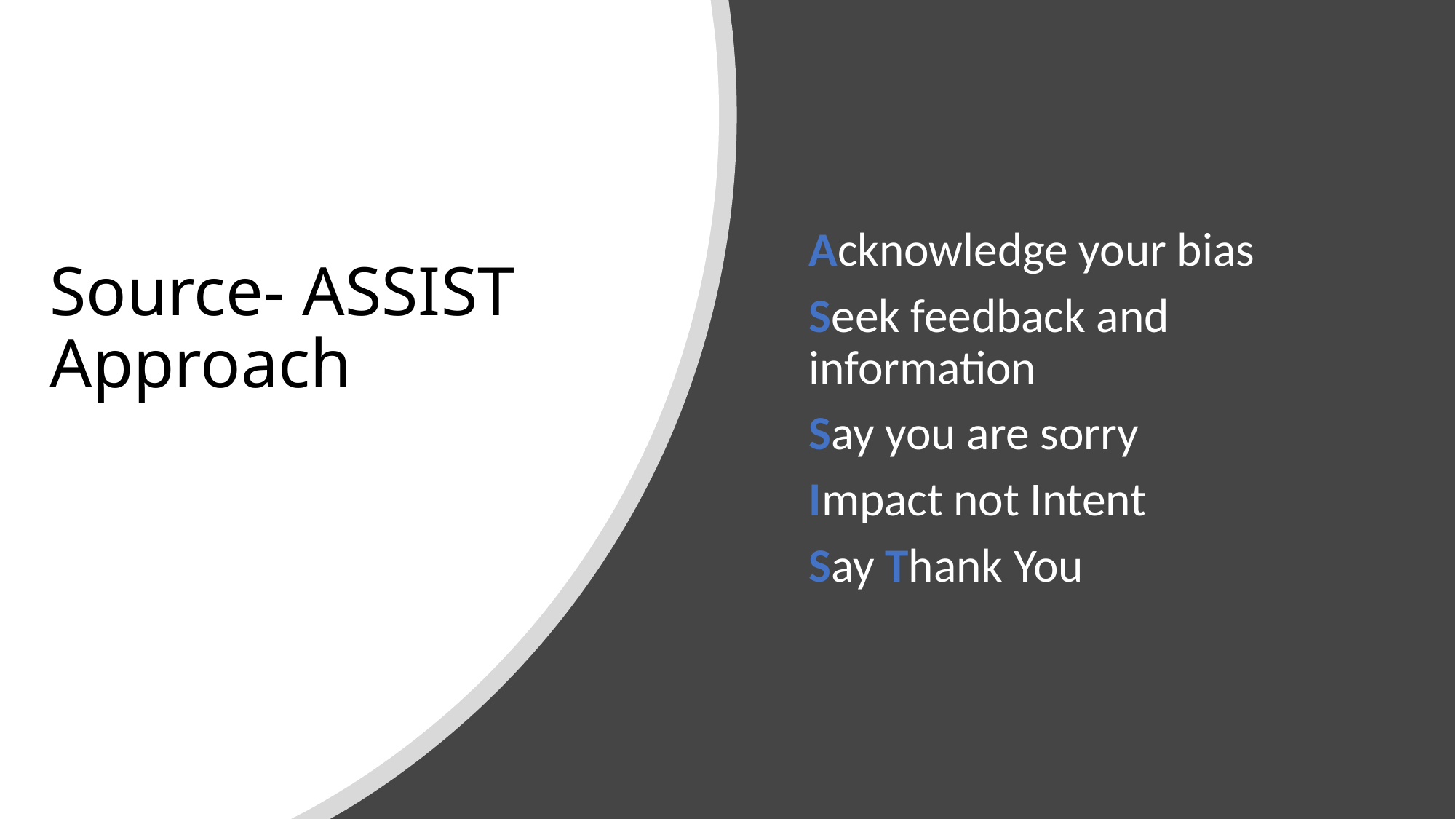

Acknowledge your bias
Seek feedback and information
Say you are sorry
Impact not Intent
Say Thank You
# Source- ASSIST Approach

## Slide 14
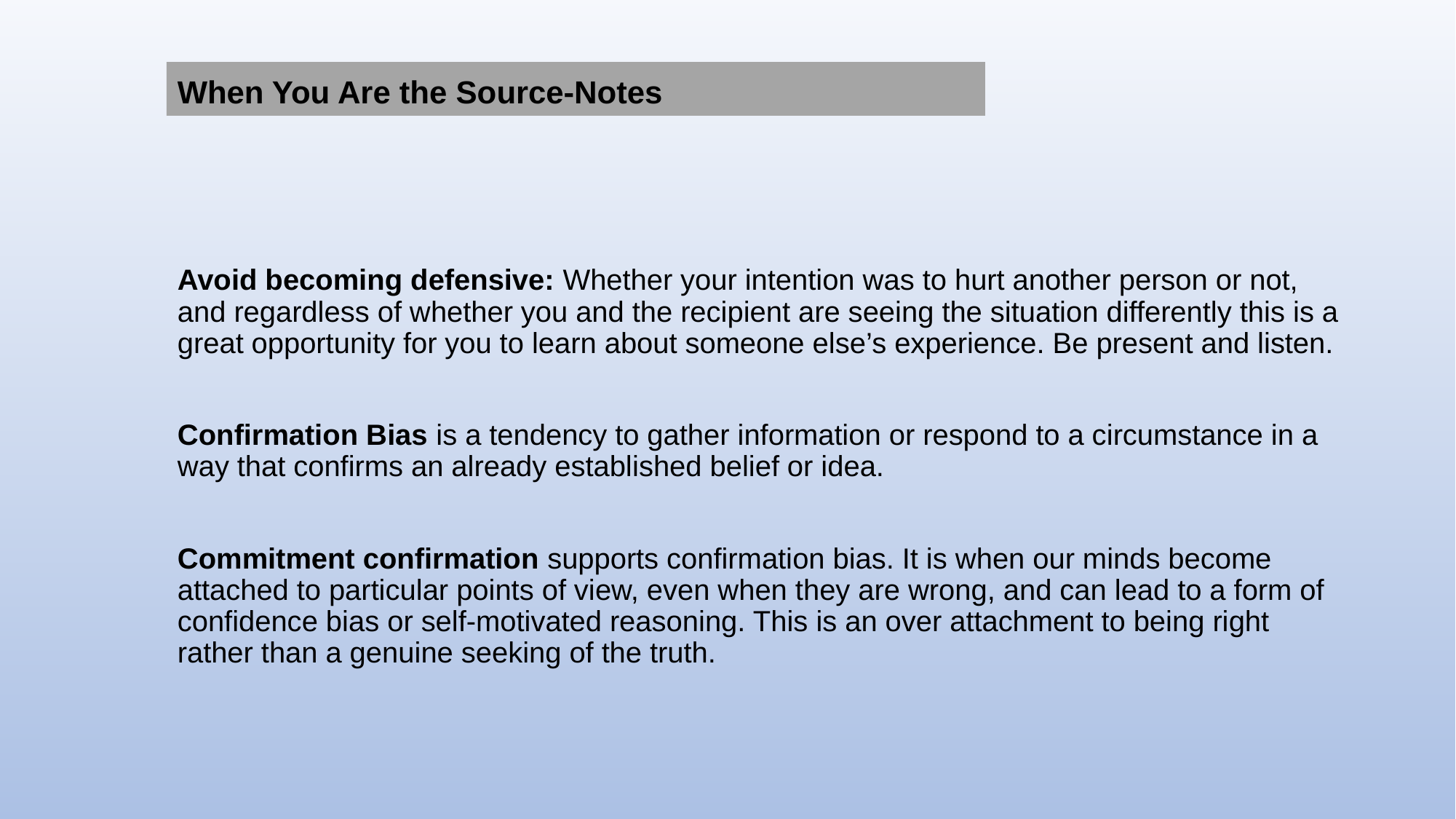

When You Are the Source-Notes
Avoid becoming defensive: Whether your intention was to hurt another person or not, and regardless of whether you and the recipient are seeing the situation differently this is a great opportunity for you to learn about someone else’s experience. Be present and listen.
Confirmation Bias is a tendency to gather information or respond to a circumstance in a way that confirms an already established belief or idea.
Commitment confirmation supports confirmation bias. It is when our minds become attached to particular points of view, even when they are wrong, and can lead to a form of confidence bias or self-motivated reasoning. This is an over attachment to being right rather than a genuine seeking of the truth.

## Slide 15
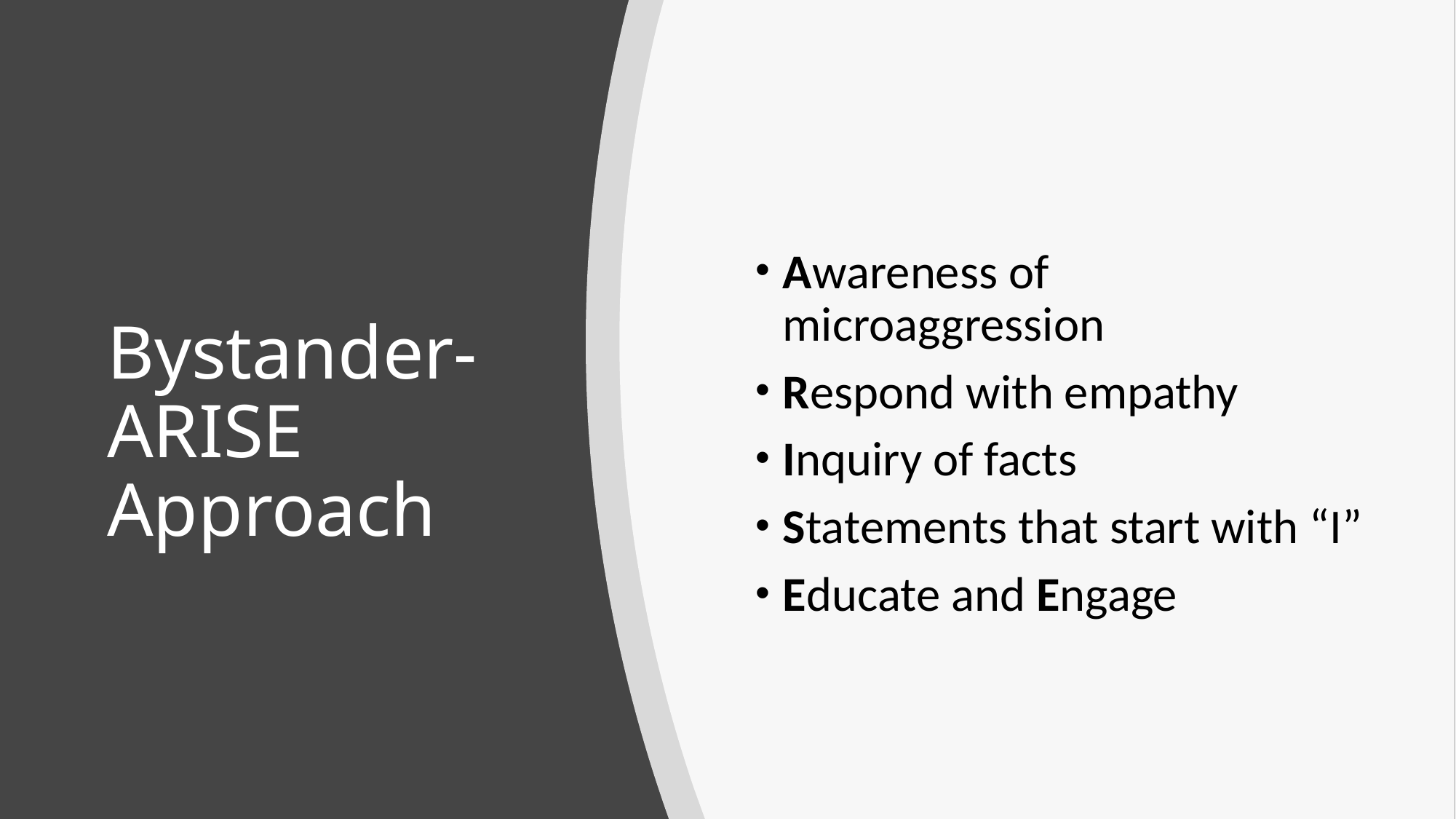

Awareness of microaggression
Respond with empathy
Inquiry of facts
Statements that start with “I”
Educate and Engage
# Bystander- ARISE Approach

## Slide 16
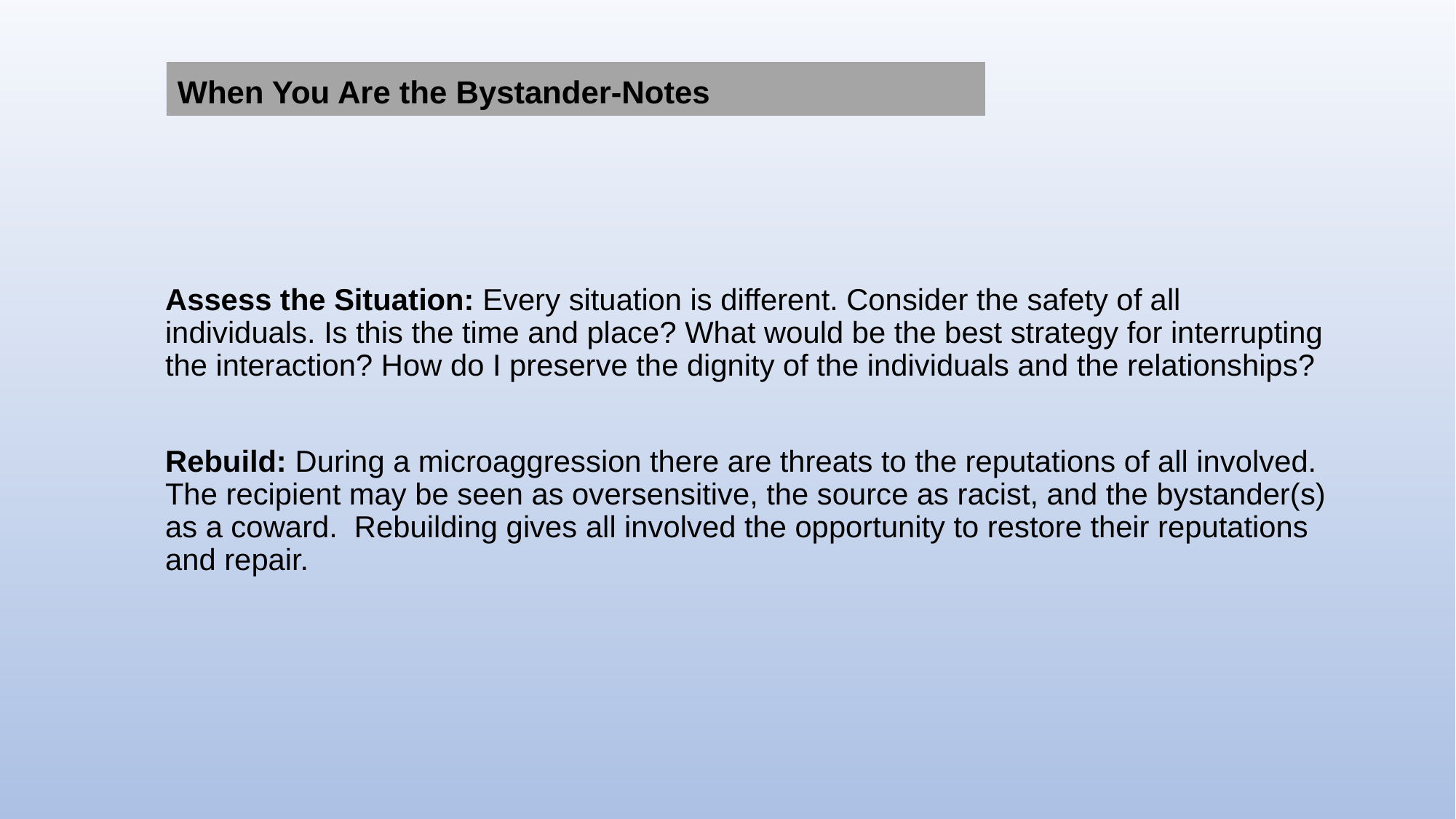

When You Are the Bystander-Notes
Assess the Situation: Every situation is different. Consider the safety of all individuals. Is this the time and place? What would be the best strategy for interrupting the interaction? How do I preserve the dignity of the individuals and the relationships?
Rebuild: During a microaggression there are threats to the reputations of all involved. The recipient may be seen as oversensitive, the source as racist, and the bystander(s) as a coward. Rebuilding gives all involved the opportunity to restore their reputations and repair.

## Slide 17
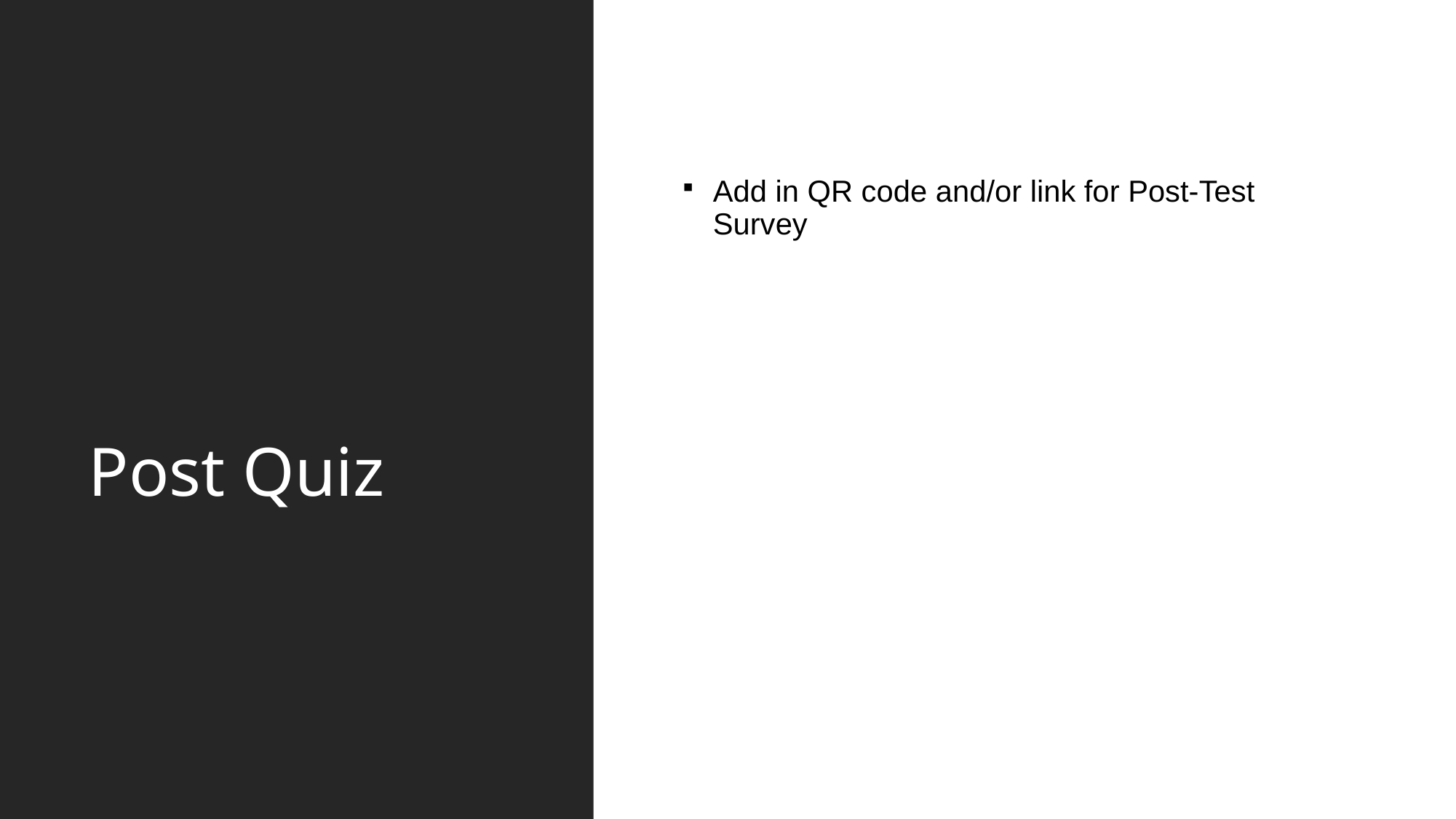

# Post Quiz
Add in QR code and/or link for Post-Test Survey
