# Supplementary material for: Addressing Microaggressions in Academic Health: A Workshop for Inclusive Excellence
Source: MedEdPORTAL. 2021 Feb 11;17:11103. doi: 10.15766/mep_2374-8265.11103 (PMC7880252; doi:10.15766/mep_2374-8265.11103)
Supplement: Supplementary file 1 — Cases & Facilitator Guides.docxPowerPoint.pptxTimetable for Learning Activities.docxHandouts for Learners.docxCore Definitions.docxPre- & Posttest.docx [file mep_2374-8265.11103-s001.zip › C. Timetable for Learning Activities.docx]

**APPENDIX C**: Timetable for Learning Activities

1. Introduction and Pre-Assessment
2. PowerPoint
3. Small Group Discussions on Case Studies (see timing below for each individual case)
4. Report Out
5. Conclusions and Post-Assessment

| PowerPoint presentation | 20-30 min |
| --- | --- |
| **Case 1-** They Haven’t Kicked You Out Yet |  |
| Scenario | 5 min |
| Discussion Guide | 20-30 min |
| **Case 2-** Taboo to Touch |  |
| Scenario | 7.5 min |
| Discussion Guide | 20-45 min |
| **Case 3–** Invite Your Husband |  |
| Scenario | 5 min |
| Discussion Guide | 20-30 min |
| **Case 4-** Don’t Worry, You’ll Get In |  |
| Scenario | 5 min |
| Discussion Guide | 20-30 min |
| **Case 5-** A Day in the Life of Female Surgeons |  |
| Scenario | 5 min |
| Discussion Guide | 20-30 min |
| **Case 6 –** Advised Away |  |
| Scenario | 5 min |
| Discussion Guide | 20-30 min |
| **Case 7-** Hidden Curriculum |  |
| Scenario | 5 min |
| Discussion Guide | 20-45 min |
