# Supplementary material for: Addressing Microaggressions in Academic Health: A Workshop for Inclusive Excellence
Source: MedEdPORTAL. 2021 Feb 11;17:11103. doi: 10.15766/mep_2374-8265.11103 (PMC7880252; doi:10.15766/mep_2374-8265.11103)
Supplement: Supplementary file 1 — Cases & Facilitator Guides.docxPowerPoint.pptxTimetable for Learning Activities.docxHandouts for Learners.docxCore Definitions.docxPre- & Posttest.docx [file mep_2374-8265.11103-s001.zip › D. Handouts for Learners.docx]

**APPENDIX D**: Handouts for Learners

I. Learner Discussion Guide

II. Cases 1-7

Learner Discussion Guide

1. Who was the recipient of the microaggression? What aspect of the recipient’s identity was the target of the microaggression?
2. Who was the source? What is the direction of the hierarchy in the relationship between the recipients and the source? For example, was the source in a position of authority, or was their position lateral or subordinate to the recipient?
3. What was the nature of the microaggression? How it could have hurtful impact on the recipient?
4. What is the historical, structural, cultural context of the microaggression?
5. How might the recipient and source be viewing the situation differently? [This section is critically important in the triangle model. Do not try to establish who is right or wrong, or what should have been said or done. Instead, focus on each person involved and, given that people are complex and not all good or all bad, try to understand what their world view may be. This helps you make an informed decision about how to proceed]

| **Recipient-ACTION Approach^1^** | **Source-ASSIST Approach** | **Bystander-ARISE Approach** |
| --- | --- | --- |
| **A**sk a clarifying question | **A**cknowledge your bias | **A**wareness of microaggression |
| **C**ome from curiosity | **S**eek feedback | **R**espond with empathy) avoid judgment) |
| **T**ell what you observed | **S**ay you are sorry | **I**nquiry of facts |
| **I**mpact Exploration | **I**mpact, not Intent | **S**tatements that start with ‘I” |
| **O**wn thoughts and feelings | **S**ay **T**hank You | **E**ducate and **E**ngage |
| **N**ext steps |  |  |

1. Discuss responses from each member involved in the interaction that could repair and re-establish relationships and restore reputations
   1. **Recipient (ACTION Approach):** Given the circumstance what are ways the recipient could address this (including not addressing it in the moment)? What can the recipient do to maintain and restore their well-being after this microaggression?
   2. **Source (ASSIST Approach):** Put yourself in the role of the source. Imagine that you become aware of your microaggression. What could you say or do to repair and re-establish relationship your relationship with the recipient (s)?
   3. **Bystander (ARISE Approach):** Whether there were bystanders or not in this scenario consider the role of a bystander or bystanders. How could they address this situation? Should it be done now or later?

- What could be done to support or protect the recipient?
- What could be said to the source that could help them become aware of the microaggression while keeping them engaged in the interaction? What are ways to help the source re-establish their relationship with the recipient (s) and restore or protect their reputation?

1. Cheung F, Ganote C, Souza T. *Microaggressions and microresistance: Supporting and empowering students* 2016.

**Case 1: They Haven’t Kicked You Boys Out Yet?**

Consider the story of an incident as retold by two different students at two different interviews, unbeknownst to the other student. Rashid and Daevion are two first year medical students. They are the only two Black men in their cohort. They each described an interaction with a White male professor.

“So, me and a fellow classmate, who is also Black, were coming in early to get some last-minute studying in before finals. And we ran into a professor that we had in Block 1. And I held the door for him, said, ‘Hi, how’s it going’ and then he’s like, ‘Oh, hi, how are you boys doing? They haven’t kicked you out yet?’ I didn’t know… was it unintentional or did it have deeper meaning? And me and my friend looked at each other and were in shock a little bit and didn’t know how to address it.

But we just went on and studied. But it was interesting because microaggressions don’t usually get to me but like I think with this one I was like, ‘Man!’, like, for 10 minutes instead of studying I was like, Ugh! Did he really just say that?” *Daevion*

“There was a time when I saw a previous professor who I consider a friend and I think he considers me a friend as well. And me and a classmate, he’s also African-American, were walking in the building. And he was like, ‘Oh, they haven’t kicked you all out yet?’ We’re friends, like, sarcasm whatever… But to somebody who isn’t like me, they could have easily been hurt by that, struck by that, triggered by that. And a bit of me was too. I was kinda like, ‘what?’”

*Rashid*

**Case 2: Taboo to Touch**

**From a Student Interview:**

There was a microaggression that almost made me fail out of school. I was doing my pediatric rotation. I had a little baby, a newborn, who was not doing well from a Hispanic or Latino family. We have this thing called “Mollera”, the front fontanelle, the soft part at the front of the head. We believe if you press on it or if you shake the baby too much, well, it’s super taboo for us to touch it. It’s part of the nursing physical assessment to palpate fontanelle. I went in with my preceptor and she did it. I was paying attention to the family because I knew what that meant for us. Since the baby was already not in the best health, their facial expression was one of almost terror- like, “Why are you touching my baby there?” But they didn’t say anything. So, when my preceptor said, “Now you do it.” I said, “you know actually … I think that’s okay. I’ll do the other things. I can explain to you about it after”. I did the rest of the assessment and when we walked out, I tried to explain to her how it was taboo and that she had already done it. I was trying to have a patient centered approach to my care. I got slammed for it. She was actually very upset with me that I had disobeyed her in front of the patient’s family. From that point on, it was just horrendous my experience with her. I almost failed that rotation.

I ended up talking to my department head. However, when I went to talk to them, they were already aware because the preceptor had called and said I wasn’t doing as I was told. I was told to suck it up. At our School of Nursing, if you fail the class, you fail the year. You repeat. There is no remediation for any clinical or any class that we do. So, the pressure is immense to pass. What ends up happening is that we get these preceptors who are very insensitive to culture and diversity. When we speak out, (I’m not the only student who has experienced this) we tell each other. The students of color at the School of Nursing warn each other about certain preceptors, about saying certain things. So, we have it as culture within our school that we warn each other because every year one minority student of the entering class fails out. It’s usually because of a preceptor and it usually because of the same preceptor all the time.

**Case 3:  Invite Your Husband**

Dr. Sheila Williams has just been hired as a new assistant professor and is meeting the other faculty at a departmental faculty meeting on her first day. Dr. Williams has just moved to town with her family and is asking the other faculty members about what to do in town with young kids. Dr. Arroyo suggests the children’s museum in town. Dr. Jensen, wishing to make her new colleague feel welcomed and included, asks Dr. Williams, “I’d like you and your husband to come over for dinner next weekend. You can bring your kids and your family can meet my family. I’m sure our husbands will get along very well.” Dr. Williams is not sure what to say because she has a wife, not a husband. She decides to take a chance and let Dr. Jensen know that she is married to a woman. Dr. Jensen responds to this by saying replies, “No need to be defensive. I’m not homophobic. I have a lot of friends who choose that lifestyle.”

**Case 4: Don’t Worry, You’ll Get In**

Dr. Wallace is leading a small group advising session for medical students who are applying for residency programs in OB/GYN. As this specialty is getting more competitive, the medical students in the group are quite anxious and are discussing their worries about whether they will be matched up with a desired program. Maria, a Latina medical student who is a first-generation college graduate, is telling the group how she applied to more residency programs than the national average because she is especially worried about not getting into this competitive specialty. Caleb, a fellow medical student who is a White male, assured Maria that she didn’t have to worry about getting into an OB/GYN residency program because she is Latina and there is a shortage of Latinxs, so she would be sure to get in as a diversity hire. Dr. Wallace noticed that Maria and several other students in the group were looking frustrated and that the tension in the room was high. Dr. Wallace didn’t say anything because she was not sure how to respond, instead she changed the topic and moved along to interviewing techniques.

**Case 5: A Day in the Life of Female Surgeons**

Juana is a 25-year-old Latina who is on her Surgery clerkship in her third year of medical school. For the past two weeks, Juana has been rotating with Dr. Linda Watson, who has inspired her to specialize in surgery and go into academic medicine. Today, she is rotating with Dr. Joaquin Hernandez, her new surgery attending, and they are scrubbing in for a case along with Dr. Arash Hakim, a surgery resident. Dr. Hernandez asks Juana what specialty she wants to go into, and Juana eagerly replies that she plans to go into surgery. Dr. Hernandez responds, “It must be really hard to be a surgeon and a mom though.” Juana talks about what a great role model Dr. Watson has been, especially around balancing her work and family life. Dr. Hernandez shrugs and replies, “Oh, I’m not saying it can’t be done, I’m just saying that every time Dr. Watson is in the OR and her girls have a soccer game or recital, she has some really tough choices to make.” Juana looks uncomfortably at Dr. Hakim and wonders to herself whether Dr. Hernandez has had any similar conversations with this resident, since fathers would likely have similar difficult decisions. Before anyone can say anything, Dr. Hernandez shrugs again and changes the topic of the conversation.

**Case 6: Advised Away**

Isabella is a Mexican-American third-year medical student, who is meeting her advisor in a group advising meeting to discuss her specialty choice and upcoming applications to residency programs. Her grades and Step 1 scores are very strong, and she has been very active in extracurricular service-based clinical activities with the Latinx community, such as leading the school’s Spanish-speaking student outreach clinic. She has developed an interest in orthopedic surgery and excitedly tells the group and advisor about her intent to apply to this specialty. Upon hearing this, the advisor shakes his head and states, “No, no, no! You can’t go into Ortho! You need to go into primary care. Your people need you!”

**Case 7: A Hidden Curriculum**

During an interview, a health professions student shared the following experience from one of their didactic classes:

“Our class went through this whole case study to review for a licensing exam. The case said,

*‘you have a patient who practices Islam and you need to figure out their nutritional needs and figure out some of their diet as a part of their care plan.’*

There were certain questions related to the case that we had to answer, one of which was *‘How do you make sure that you are being sensitive to the patient’s religion?’* While leading the class discussion the professor said, ‘It is important to make sure that if the patient is a woman that you talk to both her and her husband because that’s the way that Islam works’.”
