# Supplementary material for: Addressing Microaggressions in Academic Health: A Workshop for Inclusive Excellence
Source: MedEdPORTAL. 2021 Feb 11;17:11103. doi: 10.15766/mep_2374-8265.11103 (PMC7880252; doi:10.15766/mep_2374-8265.11103)
Supplement: Supplementary file 1 — Cases & Facilitator Guides.docxPowerPoint.pptxTimetable for Learning Activities.docxHandouts for Learners.docxCore Definitions.docxPre- & Posttest.docx [file mep_2374-8265.11103-s001.zip › E. Core Definitions.docx]

**APPENDIX E:** Core Definitions of the Toolkit

1. **Unconscious bias**) refers to the beliefs and prejudices we hold that reside outside of our awareness. Microaggressions are often the output of unconscious bias.
2. **Microaggressions** are statements, behaviors, and environmental indignities, whether intentional or unintentional, that communicate negative or denigrating racial, gender, sexual-orientation, and religious slights and insults to the target person or groups.^1,2^ This is not an exhaustive list, as microaggressions may relate to many aspects of an individual’s identity.
3. The term **Macroaggression,** according to Sue, et. al. is generally reserved to describe systemic and institutional forms of racism, sexism or homophobia that can be seen in the policy and practices of institutions like health care, judicial, education, and business and industry.^3^
4. **Inclusion** is the process by which individuals view themselves as active members of a larger community; where their background, insights and contributions are valued as part of the creativity and productivity of the group. Inclusion, then, becomes the binding force for diversity.^4^
5. **Intersectionality** is a term coined by Kimberle Crenshaw to provide a framework to explore how identity and the overlapping and interdependent forms of social stratification (such as race and gender) shape the experiences of women of color. It is since been used to understand the multiple ways that identities intersect to create very unique social experiences.^5^

1. Sue DW. *Microaggressions in everyday life: Race, gender, and sexual orientation.* John Wiley & Sons; 2010.

2. Nadal KL. The Racial and Ethnic Microaggressions Scale (REMS): Construction, Reliability, and Validity. *Journal of Counseling Psychology.* 2011;58(4):470-480.

3. Derald Wing Sue SA, Michael N. Awad, Elizabeth Glaeser, Cassandra Z. Calle and Narolyn Mendez. Disarming Racial Microaggressions: Microintervention Strategies for Targets, White Allies, and Bystanders. *American Psychologist.* 2019;74(1):128-142.

4. Ackerman-Barger K, Valderma-Wallace C, Latimore D, Drake C. Stereotype Threat Susceptibility Among Minority Health Professions Students. *Journal of Best Practices in Health Professions Diversity.* 2016;9(2):1232-1246.

5. Crenshaw K. Demarginalizing the Intersection of Race and Sex: A Black Feminist Critique of Antidiscrimination Doctrine, Feminist Theory and Antiracist Politics *University of Chicago Legal Forum* 1989.
