# Supplementary material for: Addressing Microaggressions in Academic Health: A Workshop for Inclusive Excellence
Source: MedEdPORTAL. 2021 Feb 11;17:11103. doi: 10.15766/mep_2374-8265.11103 (PMC7880252; doi:10.15766/mep_2374-8265.11103)
Supplement: Supplementary file 1 — Cases & Facilitator Guides.docxPowerPoint.pptxTimetable for Learning Activities.docxHandouts for Learners.docxCore Definitions.docxPre- & Posttest.docx [file mep_2374-8265.11103-s001.zip › F. Pre- & Posttest.docx]

**APPENDIX F**: Pre & Post Tests

*Pre-Workshop Assessment*

1. Exposure to chronic microaggressions has been shown to result in which of the following in the

recipients of microaggressions?

a. Decrement in self-esteem

b. Diminished academic performance

c. Social withdrawal

d. Anxiety and depression

e. All of the above

2. Exposure to chronic microaggressions has been shown to result in which of the following in the learning environment?

a. Recipients physically, emotionally and/or cognitively withdrawing from class

b. Diminished relationships with faculty and other students

c. Reduced inclusivity of the learning environment

d. A diversity tax for students experiencing microaggressions

e. All of the above

3. Please respond to the following statements: I feel confident in my ability to…

|  | Strongly Agree | Agree | Disagree | Strongly Disagree |
| --- | --- | --- | --- | --- |
| Notice when a microaggression is occurring |  |  |  |  |
| Respond as an active bystander when I notice a microaggression occurring |  |  |  |  |
| Respond as a recipient when I notice a microaggression occurring |  |  |  |  |
| Respond as the source when I notice I have engaged in a microaggression |  |  |  |  |
| Debrief the microaggressive situation with the recipient and other observers |  |  |  |  |

4. Name 3 strategies you could use to address microaggressions in the moment and to repair and reestablish relationships?

5. The next time I witness a microaggression occurring, I commit to being an active bystander

Strongly Agree Agree Disagree Strongly Disagree

*Post-Workshop Assessment*

1. Exposure to chronic microaggressions has been shown to result in which of the following in the

recipients of microaggressions?

a. Decrement in self-esteem

b. Diminished academic performance

c. Social withdrawal

d. Anxiety and depression

e. All of the above

2. Exposure to chronic microaggressions has been shown to result in which of the following in the learning environment?

a. Recipients physically, emotionally and/or cognitively withdrawing from class

b. Diminished relationships with faculty and other students

c. Reduced inclusivity of the learning environment

d. A diversity tax for students experiencing microaggressions

e. All of the above

3. Please respond to the following statements: I feel confident in my ability to…

|  | Strongly Agree | Agree | Disagree | Strongly Disagree |
| --- | --- | --- | --- | --- |
| Notice when a microaggression is occurring |  |  |  |  |
| Respond as an active bystander when I notice a microaggression occurring |  |  |  |  |
| Respond as a recipient when I notice a microaggression occurring |  |  |  |  |
| Respond as the source when I notice I have engaged in a microaggression |  |  |  |  |
| Debrief the microaggressive situation with the recipient and other observers |  |  |  |  |

4. Name 3 strategies you could use to address microaggressions in the moment and to repair and reestablish relationships. If you named 3 strategies before the workshop, please name 3 additional ones.

5. The next time I witness a microaggression occurring, I commit to being an active bystander

Strongly Agree Agree Disagree Strongly Disagree

6. Name one way you plan to implement the discussions today into your teaching or daily practice

7. Please rate your satisfaction with the training by responding to the statements below

|  | Strongly Agree | Agree | Neutral | Disagree | Strongly Disagree |
| --- | --- | --- | --- | --- | --- |
| The quality of the training was high |  |  |  |  |  |
| The training was relevant to my needs |  |  |  |  |  |
| I learned a new skill during this training |  |  |  |  |  |
| I would recommend this training to a colleague or classmate |  |  |  |  |  |

8. Please share any additional feedback about today’s session
