# Supplementary material for: Addressing Microaggressions in Academic Health: A Workshop for Inclusive Excellence
Source: MedEdPORTAL. 2021 Feb 11;17:11103. doi: 10.15766/mep_2374-8265.11103 (PMC7880252; doi:10.15766/mep_2374-8265.11103)
Supplement: Supplementary file 1 — Cases & Facilitator Guides.docxPowerPoint.pptxTimetable for Learning Activities.docxHandouts for Learners.docxCore Definitions.docxPre- & Posttest.docx [file mep_2374-8265.11103-s001.zip › _Educational_Summary_Report_11103.pdf]

MedEdPORTAL® The AAMC Journal of Teaching and Learning Resources

Original Publication Open Access

Addressing Microaggressions in Academic Health: A Workshop for Inclusive Excellence

Kupiri Ackerman-Barger, PhD, RN*, N. Nicole Jacobs, PhD, Regina Orozco, MSN, RN, Maya London

*Corresponding author: packerman@ucdavis.edu

Abstract

Introduction: Health profession schools have acknowledged the need for a diverse workforce by increasing diversity in recruitment, but little has been done to build inclusive excellence in learning environments. Microaggressions and other forms of mistreatment can increase stress levels and depression and negatively impact academic performance. To increase student performance, retention, and wellness, mitigating microaggressions is needed to promote an inclusive culture. **Methods:** We designed this workshop as a framework to think critically about microaggressions, how they impact the health professions academic environment, and how administrators, faculty, and students can promote inclusion excellence. The workshop included a presentation discussing microaggression theory, seven cases describing microaggressions in the health professions education environment, and discussion and facilitator guides. Cases were based on prior research conducted by the primary author and upon interactions authors shared from their professional experience. Participants completed pre- and postsurveys. **Results:** During six workshops at three different institutions, 138 out of 190 participants (73% response rate), including nursing and medicine faculty, students, and leadership, completed the pre- and postsurveys. Pre- and posttraining measurements found statistically significant improvements in participants' knowledge of the impact of microaggressions, self-efficacy in responding to microaggressions, and commitment to being an active bystander in the face of microaggressions. Participants were highly satisfied with the training. **Discussion:** This humanistic, case-based learning curriculum allows facilitators to guide faculty, student, and leadership conversations to build skills to promote inclusion excellence through preventing microaggressions, repairing and reestablishing relationships, and restoring reputations once microaggressions occur.

Keywords
Microaggression, Bystander Intervention Training, Implicit Bias, Inclusion Excellence, Racism, Discrimination, Communication Skills, Case-Based Learning, Humanistic Approach, Faculty Development, Diversity, Inclusion, Health Equity, Anti-racism

Educational Objectives

By the end of this activity, learners will be able to:

1. Identify the role of each member involved in a microaggression (recipient, source, and bystanders).
2. Discuss the nature of the microaggression, including how it could have hurtful impact on the recipients, bystanders, and/or community.
3. Analyze the historical, structural, and cultural context of the microaggression.
4. Explore how the recipient and the source may be viewing the situation differently.
5. Discuss responses from each member involved in the interaction that could build inclusive excellence, repair and reestablish relationships, and restore or protect reputations.
6. State they have increased confidence in their ability to respond to microaggressions.
7. Express a commitment to becoming active bystanders when they witness microaggressions.

Citation:
Ackerman-Barger K, Jacobs NN, Orozco R, London M. Addressing microaggressions in academic health: a workshop for inclusive excellence. *MedEdPORTAL*. 2021;17:11103.
https://doi.org/10.15766/mep_2374-8265.11103

Introduction

Microaggressions experienced by students in health professions and how to respond to these situations have become part of a discussion about ensuring that students of all backgrounds are poised to succeed in health professions schools.[1-4] The term *microaggression* describes a form of discrimination that,

Copyright © 2021 Ackerman-Barger et al. This is an open-access publication distributed under the terms of the Creative Commons Attribution license.

though often unintentional, can be communicated through verbal, nonverbal, and environmental messages.[5] Chester Pierce first used the term in 1970 to describe everyday racial slights and indignities.[6] In 2007, Sue and colleagues classified types of microaggressions (microassaults, microinsults, and microinvalidations) and expanded the definition to include any marginalized identity, such as LGBTQ or persons with disabilities.[5]

When students experience microaggressions in academic settings, their self-esteem and confidence can be depleted while their self-doubt and alienation are increased.[5,7-10] Furthermore, microaggressions can result in phenomena such as internalized racism[11] and stereotype threat, both of which can impede academic success.[12] Microaggressions in all educational settings need to be addressed for students to feel included, confident, and supported in their pursuit of a career in health professions.

Conversations about racism have become increasingly prevalent given the recent national spotlight on police brutality and violence against people of color. This workshop contributes to that discussion by offering strategies for health professions schools to train their community to respond to microaggressions in order to help create inclusive excellence at their institution. This workshop was designed to provide case-based and hands-on strategies to handle microaggressions using the Microaggressions Triangle Model framework.[4] This framework provides a humanistic approach to repairing and reestablishing relationships, as well as restoring reputations once microaggressions occur.

A review of the literature within *MedEdPORTAL* using the search terms *microaggressions, racism, inclusion, discrimination,* and *LGBT* revealed three other publications about microaggressions published up to October 2020.[13-15] These included two resources on interrupting or addressing microaggressions in the clinical setting and one providing communication tools to assist active bystanders in responding to microaggressions. The current workshop adds to that body of work by offering the Microaggressions Triangle Model,[4] which is based on helping learners to understand microaggressions from the perspectives of all parties involved, including the recipient and the bystander as well as the source of the microaggression. The workshop reviews humanistic frameworks with which all parties can respond in order to rebuild relationships and restore reputations. Furthermore, our workshop is intended for all settings (classroom, clinic, hospital, nonprofessional) with all health care audiences (nursing, medicine, physician assistants, dentists) in all roles (student, resident, faculty, leadership). Few students, faculty, or leaders receive formal training on how to recognize and respond to microaggressions with case-based opportunities to practice the responses from the perspective of the source, the recipient, and the bystander. The electronic mailing list for the AAMC Group on Diversity and Inclusion has recently seen many inquiries by members seeking tools to teach this content at their institutions. Given the primary author's expertise in microaggressions in health professions students[1] and the authors' development of the Microaggressions Triangle Model,[4] a humanistic model to respond to microaggressions, we sought to fill the gap in the literature with this workshop. It was first created to be used at monthly lunch-and-learn diversity dialogues as a method for helping medical students to recognize and understand the nature of microaggressions and how to respond to them. It was then delivered more broadly to other health professions audiences and roles of participants (students, faculty, leadership) and in multiple settings, as described in the Methods section. The workshop expands upon our Microaggressions Triangle Model by providing case-based scenarios and a facilitator's guide.

Methods

This workshop focused on health professions academic situations in which microaggressions occur. Drawing upon examples from the first author's study of microaggressions in health professions schools,[1] we developed seven case scenarios illustrative of the types of microaggressions health professions learners and faculty might experience in academic and clinical settings. The case scenarios were designed specifically for members of the health professions academic community, including faculty, leadership, staff, and students. However, users from diverse professional backgrounds will find material that is useful in understanding microaggressions, learning how to respond to microaggressions, and promoting inclusion in their settings. Learning activities included a PowerPoint presentation on microaggressions and the Microaggressions Triangle Model,[4] case scenarios, and small-group discussions. The case scenarios included in the activities were based on interactions that the authors shared from their professional experience and on research from several studies conducted by the primary author.[1] The discussion guides were informed by literature in psychology, critical race theory in education, intersectionality, nursing, and academic medicine.

Each scenario was reviewed by multiple health professions audiences, including nursing, medicine, and physician assistants, who were students, faculty, medical residents, or graduate students. These individuals represented a wide range of racial/ethnic backgrounds, ages, and genders, and included members of the LGBT community. These reviews added depth to the discussion guide and helped the authors see past their

Copyright © 2021 Ackerman-Barger et al. This is an open-access publication distributed under the terms of the Creative Commons Attribution license.

own individual lenses. We then developed a facilitator's guide to highlight the nature of the microaggressions in each case, how the microaggressions could be rooted in a history of bias and systemic racism, the roles of all participants in the case interaction, and how each participant could respond using the Microaggressions Triangle Model,[4] with opportunities for workshop participants to behaviorally practice applying the model to the cases. In order to assess the impact of the workshop, we developed pre- and posttest assessments. Using Kirkpatrick's hierarchy of levels of learning evaluation,[16] these assessments were designed to reflect the objectives of the workshop and to analyze change in participants' knowledge about microaggressions (Kirkpatrick level 2, learning), self-efficacy in responding to microaggressions, and commitment to being an active bystander in the face of microaggressions (Kirkpatrick level 3, behavioral change). We piloted the assessments in December 2019 with a group of psychiatry residents at the University of California, Davis, and then revised the assessments for usability and readability. We also added questions to assess satisfaction with the workshop (Kirkpatrick level 1, reaction).

The seven scenarios in this workshop could be used independently, as a series of cases, or as a daylong intensive training (Appendix A). The PowerPoint presentation (Appendix B) accompanying the cases was intended to take 20-30 minutes to complete. Each case took 20-45 minutes depending on its complexity as well as on the number and level of engagement of the learners (Appendix C).

Prework for Facilitators and Learners

The Microaggressions Triangle Model was developed by Ackerman-Barger and Jacobs as a framework for understanding microaggressions from a humanistic standpoint.[4] The model provided a framework with acronyms to help individuals respond to microaggressions whether they were the recipient, the source, or the bystander (Figure 1). Both facilitators and learners were asked to read an article on this model[4] prior to holding the workshop. The scenarios and discussions in the workshop were designed to get learners to put themselves in the shoes of each person in the Microaggression Triangle Model and to practice applying the ACTION,[17] ARISE, and ASSIST approaches for each character in the scenario.

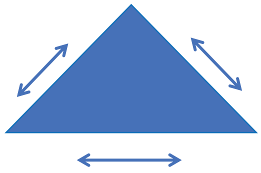

Figure 1. The Microaggressions Triangle Model.

- Recipient—ACTION approach17:
    - A: Ask a clarifying question.
    - C: Come from curiosity.
    - T: Tell what you observed.
    - I: Impact exploration.
    - O: Own thoughts and feelings.
    - N: Next steps.
- Source—ASSIST approach:
    - A: Acknowledge your bias.
    - S: Seek feedback.
    - S: Say you are sorry.
    - I: Impact, not intent.
    - ST: Say thank you.
- Bystander—ARISE approach
    - A: Awareness of microaggression.
    - R: Respond with empathy.
    - I: Inquiry of facts.
    - S: Statements that start with "I."
    - E: Educate and engage.

Implementing the Activity

We presented the workshop on six different occasions to a total of 190 individuals (students and employees [including faculty, staff, and leadership]) representing both the nursing and medical professions from three universities (University of California, Davis; University of Nevada, Reno; and University of Portland). Workshops were delivered to participants from different disciplines separately, and not in an interprofessional learning environment. Workshops were conducted in person prior to the COVID-19 pandemic and were provided online (via Zoom) starting in March 2020. For in-person workshops, we utilized a large room with a computer set up to show the PowerPoint presentation and then asked smaller groups to move to various parts of the room to discuss the cases. For online sessions, the PowerPoint presentation was shared with the large group all together, and then, breakout rooms were used to host the small-group discussions of the cases.

Learners were given the Microaggressions Triangle Model article[4] and asked to read it prior to the workshop. During the workshop, learners were given handouts (Appendix D), including the learner discussion guide and the cases. To ensure that learners understood nuanced terminology related to microaggressions and inclusion, workshop participants were also provided with

Copyright © 2021 Ackerman-Barger et al. This is an open-access publication distributed under the terms of the Creative Commons Attribution license.

definitions (Appendix E). Faculty leading the small-group case discussions were given all appendices and asked to familiarize themselves with definitions, the Microaggressions Triangle Model, the cases, and the facilitator's guide prior to the small-group sessions.

We began the activity by discussing the purpose of the workshop and having learners read the learning objectives so that they knew what to expect. Learners were then asked to take a pretest assessment (Appendix F) to help us understand the impact of the workshop. Next, the PowerPoint presentation describing microaggressions and containing more in-depth explanations for how to implement the ACTION,[17] ASSIST, and ARISE responses was reviewed in a large-group setting by one facilitator.

Before the learners broke into small groups to work through the cases, the large-group facilitator discussed guidelines for a safe and respectful learning environment in the small groups to ensure that all members of a group felt safe in sharing and asking questions. Reviewing group guidelines was especially important since the purpose of the case discussion was not to expose which students had experienced microaggressions but to learn and grow collectively. Small-group facilitators were instructed to use a humanistic approach to guide the students and to model what we would be teaching.

The large group was then broken down into smaller groups, with an ideal size of eight to 12 learners. Small-group facilitators reinforced the agreements for engagement previously laid out in the large-group discussion. Before discussing any of the cases, we began with a low-stakes icebreaker. For example, we had group members share something about themselves that was unique and that people would not know by looking at them. We then transitioned to the cases, emphasizing that the purpose of discussing them was not to expose those who might have unconscious bias but rather to learn and grow collectively, as discussions about race, gender, and marginalized identities can be emotionally charged interactions.

We instructed learners to read the scenario independently and have one participant read it out loud to the group. We also asked learners to write their thoughts on a sheet of paper or on their devices. This gave them time for private reflection. After the private reflection, we structured discussion in several ways depending on the time frame and the particular group of learners. In paired or small-group discussion, we asked learners to work through cases with a partner or group of partners. This gave them time to verbally work through their thoughts and reflections without the pressure of the entire class listening.

After the small groups discussed the cases, we brought the entire class together again in the large-group setting. Participants were asked to share important lessons learned from each case reviewed. We utilized one of three ways to sum up the cases, depending upon time limits and the size of the group:

1. For smaller groups of up to 12 participants, we went through each question with the group to garner the perspectives of every participant and to uncover nuances. We allotted additional time for using this approach (20-25 minutes).
2. For medium-sized groups of 13-70, we used a couple of targeted questions about the scenario and asked the small groups to briefly report out on their insights (see the key points in each case in Appendix A for potential questions). We allotted 15 minutes per case when using this approach.
3. For large groups of over 70 participants, we had the instructor summarize key points from each scenario (see the key points in each case in Appendix A for a summary). This approach took 5-10 minutes per case.

At the end of the workshop, facilitators summarized key points in order to reinforce learning. Finally, participants were asked to take a posttest (Appendix F), which featured the same questions as the pretest on knowledge, self-efficacy, and commitment to action, as well as including questions assessing participants' satisfaction with the workshop.

Analysis of the Impact of Workshop

To evaluate the impact of the workshop, we offered participants a pretest at the beginning of each workshop and a posttest immediately following each workshop. We received institutional review board approval from both the University of California, Davis, and the University of Nevada, Reno. We administered surveys via Qualtrics, using a QR code or link so that participants could access the surveys on their mobile phones or laptops. The survey was designed to measure knowledge, self-efficacy, commitment, and satisfaction. Participants at the University of Portland did not receive the satisfaction questions because they were the first to undergo the workshop and satisfaction questions had not yet been developed. Knowledge was measured by two multiple-choice questions about the impact of microaggressions and an open-response question on strategies to address microaggressions (Appendix F, questions 1, 2, and 4). Self-efficacy, defined as a belief in one's capability to respond to and manage prospective situations, was measured as the average response on five 4-point Likert-style statements (1 = *strongly agree,* 4 = *strongly disagree*; Appendix F,

Copyright © 2021 Ackerman-Barger et al. This is an open-access publication distributed under the terms of the Creative Commons Attribution license.

question 3). Commitment to becoming an active bystander was measured using one 4-point Likert scale question (Appendix F, question 5). The postsurvey included all the presurvey questions as well as an additional four questions about satisfaction with the workshop, an open-response question assessing how participants planned to implement what was learned at their home institutions, and the opportunity to provide comments, questions, or recommendations regarding the workshop.

We compiled survey responses from participants in all sessions and analyzed them in the aggregate. Data were also analyzed by role to determine if there was a differential impact for types of learners. Change from pre- to posttraining in self-efficacy, knowledge, and commitment was tested using a Wilcoxon signed rank test (the nonparametric paired *t*-test equivalent). We used R Studio, version 1.2.5019, for statistical analyses.[17] To minimize the chances of Type 1 error given multiple analyses, we used a Bonferroni correction and thus, in all cases, assessed significance at $\alpha = .017$.

Results

Pre- and postsurveys were completed by 138 out of 190 participants, for an overall response rate of 73%. Summary statistics for knowledge are presented as percentage of correct answers, while Likert-scale data are presented as mean and standard deviation for all other measures. Data from all participants were analyzed in the aggregate, and then, post hoc analyses were conducted by role (student and employee [including faculty, staff, and leadership combined]) to see if there were differential effects on students versus employees. Most participants answered the knowledge questions correctly both before ($M = 95\%$, $SD = 18\%$) and after ($M = 99\%$, $SD = 10\%$) the training, with a statistically significant 4% improvement from pre- to postworkshop ($V = 11.0$, $p = .015$; Table). When analyzed by role, students experienced a small but statistically significant ($V = 11.0$, $p = .015$; Table, Figure 2) 6% increase in knowledge from before ($M = 92\%$, $SD = 22\%$) to after ($M = 98\%$, $SD = 12\%$) the training, and employees started at 100% correct prior to the training and maintained that knowledge at 100% after the training.

Prior to the training, the mean self-efficacy score for all participants was 2.74 ($SD = 0.49$), suggesting that on average, the participants did not agree that they felt confident in their ability to respond to microaggressions they witnessed and/or experienced (Table, Figure 2). Following the training, participants' confidence in their ability to respond to microaggressions increased by 24% ($M = 3.41$, $SD = 0.53$), showing significantly higher overall confidence ($V = 79.5$, $p < .001$) and falling above the agreement threshold (Table, Figure 2). Post hoc analyses examining self-efficacy by role indicated significant improvements ($V = 40.0$, $p < .001$) from pre- ($M = 2.70$, $SD = 0.5$) to posttraining ($M = 3.45$, $SD = 0.56$) for students as well as employees (pre $M = 2.81$, $SD = 0.50$; post $M = 3.33$, $SD = 0.45$; $V = 3.5$; $p < .001$; Table, Figure 2). Self-efficacy among students and employees increased by 28% and 18%, respectively.

Table. Summary Statistics and Results of Wilcoxon Signed Rank Tests

Role and Variables	*M* (*SD*) Pretraining	*M* (*SD*) Posttraining	V	*p*
Overall (*N* = 138)				
Knowledge	94.9 (18.41)	98.9 (9.49)	11.0	.015[a]
Self-efficacy	2.7 (0.49)	3.4 (0.53)	79.5	<.001[a]
Commitment	3.3 (0.67)	3.6 (0.58)	140.0	<.001[a]
Satisfaction[b]		3.7 (0.51)		
Students (*N* = 90)				
Knowledge	92.2 (22.37)	98.3 (11.73)	11.0	.015[a]
Self-efficacy	2.7 (0.50)	3.5 (0.56)	40.0	<.001[a]
Commitment	3.3 (0.73)	3.7 (0.62)	70.0	<.001[a]
Satisfaction		3.7 (0.54)		
Employees (*N* = 48)				
Knowledge	100.0 (0.00)	100.0 (0.00)	0.0	
Self-efficacy	2.8 (0.46)	3.3 (0.45)	3.5	<.001[a]
Commitment	3.5 (0.50)	3.6 (0.49)	13.0	.023
Satisfaction[c]		3.9 (0.28)		

[a]Statistically significant.
[b]Posttraining *N* = 105.
[c]Posttraining *N* = 15.

The single-item assessment of commitment also increased by 8% from before ($M = 3.33$, $SD = 0.67$) to after ($M = 3.58$, $SD = 0.58$) the training ($V = 140.0$, $p < .001$; Table, Figure 2). Post hoc analyses by role showed a 12% increase in commitment from pre- ($M = 3.27$, $SD = 0.73$) to posttraining ($M = 3.67$, $SD = 0.62$) for students that was statistically significant ($V = 70.0$, $p < .001$). Employees started with a higher level of commitment than students pretraining ($M = 3.45$, $SD = 0.50$) but showed a 4% improvement at posttraining ($M = 3.60$, $SD = 0.49$) that was a trend given the Bonferroni corrections ($V = 13.0$, $p = .023$).

Of the 138 respondents, 33 participants from the University of Portland, who were all faculty, did not receive the satisfaction questions. Among those who did ($N = 105$), overall mean satisfaction was very high ($M = 3.72$, $SD = 0.51$; Table, Figure 2). When analyzed by role, satisfaction remained high for students ($M = 3.70$, $SD = 0.54$) and was slightly higher for employees ($M = 3.88$, $SD = 0.28$).

Discussion

Knowing the prevalence and detrimental impact of microaggressions on health professions learners and faculty, we developed a workshop to teach participants to recognize

Copyright © 2021 Ackerman-Barger et al. This is an open-access publication distributed under the terms of the Creative Commons Attribution license.

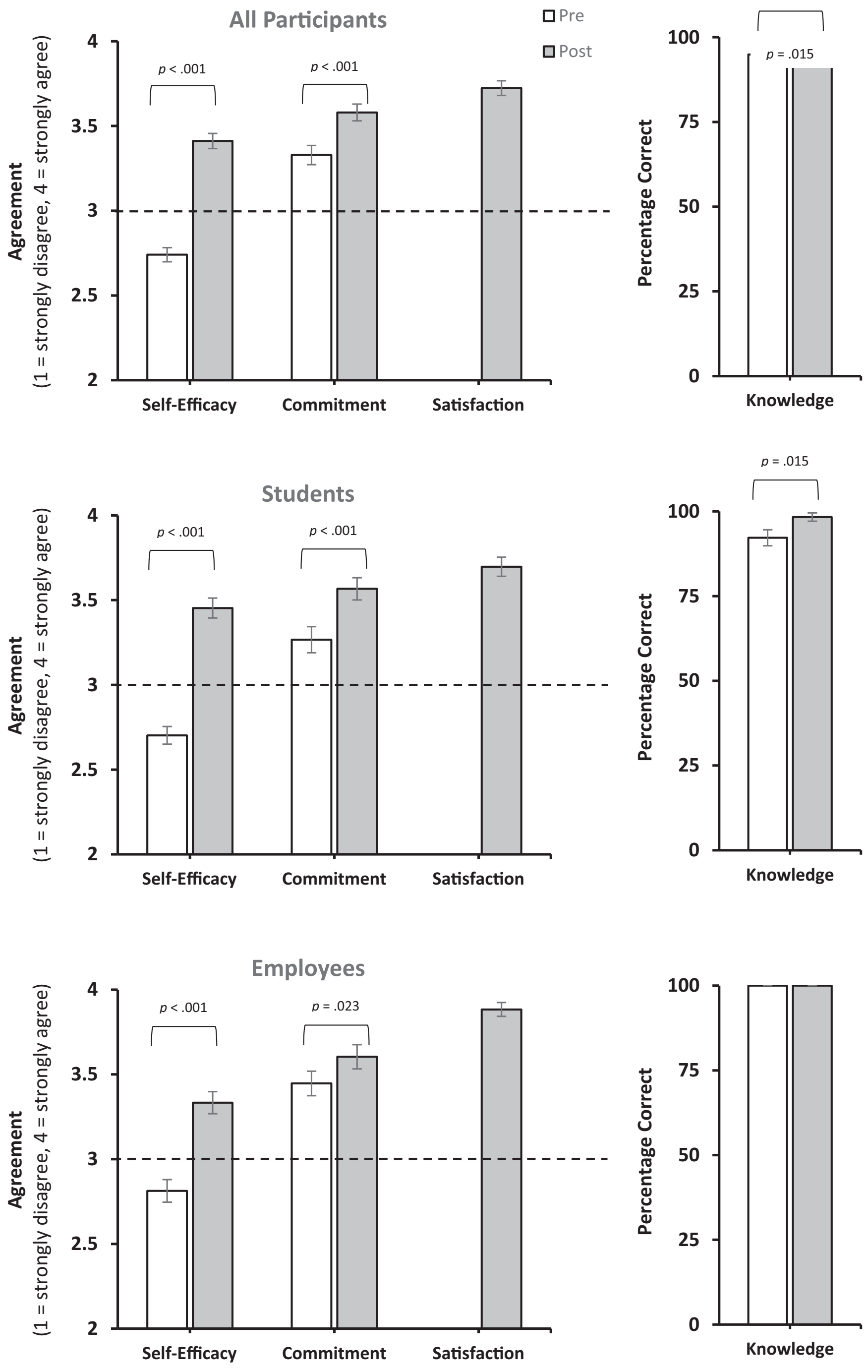

Figure 2. Mean values and standard error bars for participant responses to questions related to self-efficacy, commitment, satisfaction, and knowledge, from before (white) and after (gray) the microaggressions training. The dotted line shows the threshold for "agree."

and respond to microaggressions in a manner intended to create teachable moments, promote allyship, and advance inclusive excellence in health profession schools. Survey results comparing pre/post data for participants completing the training showed a statistically significant improvement in participants' knowledge of the impact of microaggressions, self-efficacy in responding to microaggressions, and commitment to being an active bystander in the face of microaggressions. The high rate of satisfaction reported by participants suggests that this format is appealing. This satisfaction is particularly important

Copyright © 2021 Ackerman-Barger et al. This is an open-access publication distributed under the terms of the Creative Commons Attribution license.

given the sensitive nature of discussing microaggressions and the fact that participation was mandatory for students. One of the strengths of the workshop is the humanistic framework upon which it is built. In this approach, rather than calling people out for racist behaviors and assigning blame, we teach participants to call people in to build relationships and empathy. The Microaggressions Triangle Model encourages people to view microaggressions from all perspectives and humanizes individuals by encouraging participants to step into the shoes of others, whether they are the recipient, the source, or the bystander.[4] This provides a less threatening way to engage in the difficult conversations that can promote inclusion.

The approaches used in this workshop are grounded in mindfulness and perspective-taking, which have been shown to manage implicit bias and foster empathy.[18-20] Mindfulness is the ability to be present in the moment and to notice thoughts, physical sensations, and environmental cues.[18] It is through mindfulness that people can both become aware of their unconscious biases and align their behaviors with their values. Perspective-taking is the ability to see the world from the perspective of others, thereby increasing empathy.[20] By looking at the three different perspectives of the Microaggressions Triangle Model and seeing themselves in all three of those roles, people can develop the skill of perspective-taking.[4] This humanistic grounding in mindfulness and perspective-taking can result in interventions for microaggressions that are truly focused on rebuilding relationships, restoring reputations, and building community. Perspective-taking also helps people explore and recommit to their individual and collective values.

Another strength of the workshop is that because we surveyed 138 individuals representing different disciplines (nursing and medicine) and various roles (health professions students, faculty, staff, and leadership) across three universities, it is likely this tool will be useful in many settings and with a variety of audiences, including interprofessional learners The workshop includes a full package of learning with interactive components based on adult learning principles. The case scenarios allow participants to engage authentically with interactions without having to share their own experiences. This lets them be more objective and cognitively present during discussions while minimizing the risk of harm from having personal stories be invalidated. Byrne and Tanesini have made the case that individuals can learn new associations through repeated opportunities to reflect upon and act out their values.[21] The workshop's seven case scenarios give participants multiple opportunities to reflect upon their values and to learn skills to enact them. With high satisfaction ratings, the training has proven to be feasible, even if implemented as a mandatory training. It can be delivered in a flexible manner, either in person in a classroom setting or online using a virtual platform that allows for breakout rooms for small-group discussion.

There were lessons learned in implementing this workshop, especially since we were suddenly forced to move from in-person workshops to virtual ones via videoconferencing due to the COVID-19 pandemic. When we developed the workshop, we envisioned face-to-face sessions that would foster interaction and connection among learners. As we transitioned to an online format, there were challenges, such as eliciting engagement and connection among participants when some learners muted their audio and video and engaged less verbally. In general, although we received positive evaluations, we found that there were variations in the degree to which learners engaged. At times, this required instructors to be nimble and to decide, in real time, whether it was better to have in-depth conversations about fewer cases or more surface conversations about many cases. We also grappled with decisions related to how much facilitation was needed in small groups: Because discussions about microaggressions can result in differing opinions or points of view, some degree of conflict could occur and, without facilitation, could result in harm versus learning and growth. When we did not have enough facilitators trained in the Microaggressions Triangle Model available, we assigned volunteer small-group facilitators or did not break out into small groups but kept everyone in a large group, with the entire discussion facilitated by the instructor. We found that this was an option that worked well, especially if the group was smaller than 20 participants.

There were some limitations to the study of the workshop. In the domain of knowledge, only two questions were asked to assess knowledge about the impact of microaggressions, and they were not difficult enough, which created a ceiling effect even at pretraining. In addition, our knowledge questions focused on the impact of microaggressions rather than the context of microaggressions. This made it difficult to assess whether change in knowledge occurred as a result of the training. Another limitation was that many employees self-selected to participate in the study, meaning they may have already had knowledge of and/or commitment to addressing microaggressions. This may or may not have impacted our results related to whether the training influenced commitment to addressing microaggressions. Furthermore, because the sample included many self-selected participants, we may not have adequately captured the perceptions of people required to participate in a workshop on addressing microaggressions. Although we were able to assess

Copyright © 2021 Ackerman-Barger et al. This is an open-access publication distributed under the terms of the Creative Commons Attribution license.

the workshop across professions, our only opportunities for interprofessional trainings were small groups during the phase when we were pilot-testing individual cases. This meant that we were not able to formally survey participants in interprofessional workshops. There were also many microaggressions that we did not capture in the seven case scenarios, including disability, weight, age, and more. It should not be inferred that microaggressions unrepresented in our case scenarios are not important. Our workshop is meant only as a starting place to explore how to address microaggressions, no matter what form they take. Finally, we did not evaluate whether people's behavior changed over an extended period of time. A longitudinal study could establish whether participants continue to experience self-efficacy and commitment to addressing microaggressions in the long term.

As the United States grapples with the depth and breadth of racial and social injustices in so many of its institutions, including health care, many people want to learn more about the history of racism and injustice and are asking what they can do to promote inclusion and uphold principles of a just society. This workshop addresses microaggressions from a humanistic perspective and, therefore, is a great place to start. We provide historical, structural, and contextual information about the nature of the microaggressions to create teachable moments and to help learners connect both intellectually and emotionally with the impact microaggressions can have on individuals and the community. In addition, we also provide skills training and opportunities for practice on how to respond to microaggressions from all perspectives.

Conversations about how to address microaggressions and promote inclusion in health professions academic settings are necessary for both student and employee wellness, to create inclusive excellence in academic environments, and to heal the country. Although there are many approaches to promoting inclusion, we offer our workshop as an engaging package of learning tools applicable in multiple settings. Whatever format schools use, dialoging and skill building are essential components of increasing inclusion excellence.

Appendices

A. Cases & Facilitator Guides.docx

B. PowerPoint.pptx

C. Timetable for Learning Activities.docx

D. Handouts for Learners.docx

E. Core Definitions.docx

F. Pre- & Posttest.docx

All appendices are peer reviewed as integral parts of the Original Publication.

Kupiri Ackerman-Barger, PhD, RN: Associate Dean of Health Equity, Diversity and Inclusion, Betty Irene Moore School of Nursing at University of California, Davis; Co-Director, Center for a Diverse Healthcare Workforce, University of California, Davis, School of Medicine

N. Nicole Jacobs, PhD: Associate Dean of Diversity and Inclusion, Office of Diversity and Inclusion, University of Nevada, Reno, School of Medicine; ORCID: https://orcid.org/0000-0003-2697-3911

Regina Orozco, MSN, RN: Doctoral Student, Betty Irene Moore School of Nursing at University of California, Davis

Maya London: Junior Specialist, Center for a Diverse Healthcare Workforce, University of California, Davis, School of Medicine; ORCID: https://orcid.org/0000-0002-4422-9953

Disclosures
None to report.

Funding/Support
None to report.

Ethical Approval
The University of California, Davis, Institutional Review Board and the University of Nevada, Reno, Institutional Review Board approved this study.

Disclaimer
This work is supported by the Health Resources and Services Administration (HRSA) of the U.S. Department of Health and Human Services (HHS) as part of an award totaling $3,791,026, with 0% financed with nongovernmental sources. The contents are those of the authors and do not necessarily represent the official views of, nor an endorsement by, HRSA, HHS, or the U.S. government. For more information, please visit HRSA.gov.

References

1. Ackerman-Barger K, Boatright D, Gonzalez-Colaso R, Orozco R, Latimore D. Seeking inclusion excellence: understanding racial microaggressions as experienced by underrepresented medical and nursing students. *Acad Med*. 2020;95(5):758-763. https://doi.org/10.1097/ACM.0000000000003077
2. Espaillat A, Panna DK, Goede DL, Gurka MJ, Novak MA, Zaidi Z. An exploratory study on microaggressions in medical school: what are they and why should we care? *Perspect Med Educ*. 2019;8(3):143-151. https://doi.org/10.1007/s40037-019-0516-3

Copyright © 2021 Ackerman-Barger et al. This is an open-access publication distributed under the terms of the Creative Commons Attribution license.

3. Ulloa JG, Talamantes E, Moreno G. Microaggressions during medical training. *JAMA*. 2016;316(10):1113-1114. https://doi.org/10.1001/jama.2016.11619

4. Ackerman-Barger K, Jacobs NN. The Microaggressions Triangle Model: a humanistic approach to navigating microaggressions in health professions schools. *Acad Med*. 2020;95(12)(suppl): S28-S32. https://doi.org/10.1097/ACM.0000000000003692

5. Sue DW, Capodilupo CM, Torino GC, et al. Racial microaggressions in everyday life: implications for clinical practice. *Am Psychol*. 2007;62(4):271-286. https://doi.org/10.1037/0003-066X.62.4.271

6. Pierce C. *Offensive mechanisms*. In: Barbour FB, ed. *The Black Seventies*. Porter Sargent; 1970:265-282.

7. Nadal KL, Wong Y, Griffin KE, Davidoff K, Sriken J. The adverse impact of racial microaggressions on college students' self-esteem. *J Coll Stud Dev*. 2014;55(5):461-474. https://doi.org/10.1353/csd.2014.0051

8. Solorzano D, Ceja M, Yosso T. Critical race theory, racial microaggressions, and campus racial climate: the experiences of African American college students. *J Negro Educ*. 2000;69(1-2): 60-73.

9. Allen Q. Racial microaggressions: the schooling experiences of Black middle-class males in Arizona's secondary schools. *J Afr Am Males Educ*. 2010;1(2):125-143.

10. Pulliam N, Sasso P, Paone TR, Maldonado JM. Reducing systemic racism: movements toward change in higher education. In: Stead V, ed. *RIP Jim Crow: Fighting Racism Through Higher Education Policy, Curriculum, and Cultural Interventions*. Peter Lang Publishing; 2016:129-148.

11. Kohli R, Solórzano DG. Teachers, please learn our names!: racial microaggressions and the K-12 classroom. *Race Ethn Educ*. 2012;15(4):441-462. https://doi.org/10.1080/13613324.2012.674026

12. Steele CM. *Whistling Vivaldi: How Stereotypes Affect Us and What We Can Do*. W. W. Norton; 2010.

13. Acholonu RG, Cook TE, Roswell RO, Greene RE. Interrupting microaggressions in health care settings: a guide for teaching medical students. *MedEdPORTAL*. 2020;16:10969. https://doi.org/10.15766/mep_2374-8265.10969

14. Sandoval RS, Afolabi T, Said J, Dunleavy S, Chatterjee A, Ölveczky D. Building a tool kit for medical and dental students: addressing microaggressions and discrimination on the wards. *MedEdPORTAL*. 2020;16:10893. https://doi.org/10.15766/mep_2374-8265.10893

15. Sotto-Santiago S, Mac J, Duncan F, Smith J. "I didn't know what to say": responding to racism, discrimination, and microaggressions with the OWTFD approach. *MedEdPORTAL*. 2020;16:10971. https://doi.org/10.15766/mep_2374-8265.10971

16. Kurt S. Kirkpatrick model: four levels of learning evaluation. Educational Technology. October 4, 2016. Updated September 6, 2018. https://educationaltechnology.net/kirkpatrick-model-four-levels-learning-evaluation/

17. Cheung F, Ganote C, Souza T. Microaggressions and microresistance: supporting and empowering students. In: *Diversity and Inclusion in the College Classroom*. Magna Publications; 2016:15-17.

18. Burgess DJ, Beach MC, Saha S. Mindfulness practice: a promising approach to reducing the effects of clinician implicit bias on patients. *Patient Educ Couns*. 2017;100(2):372-376. https://doi.org/10.1016/j.pec.2016.09.005

19. Lueke A, Gibson B. Mindfulness meditation reduces implicit age and race bias: the role of reduced automaticity of responding. *Soc Psychol Personal Sci*. 2015;6(3):284-291. https://doi.org/10.1177/1948550614559651

20. Todd AR, Galinsky AD. Perspective-taking as a strategy for improving intergroup relations: evidence, mechanisms, and qualifications. *Soc Personal Psychol Compass*. 2014;8(7):374-387. https://doi.org/10.1111/spc3.12116

21. Byrne A, Tanesini A. Instilling new habits: addressing implicit bias in health care professionals. *Adv Health Sci Educ Theory Pract*. 2015;20(5):1255-1262. https://doi.org/10.1007/s10459-015-9600-6

Received: June 12, 2020
Accepted: November 19, 2020
Published: February 11, 2021

Copyright © 2021 Ackerman-Barger et al. This is an open-access publication distributed under the terms of the Creative Commons Attribution license.
